# A systematic pan-cancer study on deep learning-based prediction of multi-omic biomarkers from routine pathology images

**DOI:** 10.1038/s43856-024-00471-5

**Published:** 2024-03-15

**Authors:** Salim Arslan, Julian Schmidt, Cher Bass, Debapriya Mehrotra, Andre Geraldes, Shikha Singhal, Julius Hense, Xiusi Li, Pandu Raharja-Liu, Oscar Maiques, Jakob Nikolas Kather, Pahini Pandya

**Affiliations:** 1 Panakeia Technologies, London, UK; 2 Department of Pathology, Barking, Havering and Redbridge University NHS Trust, Romford, UK; 3 Department of Pathology, The Royal Wolverhampton NHS Trust, Wolverhampton, UK; 4 Cytoskeleton and Cancer Metastasis Group, Breast Cancer Now Toby Robins Breast Cancer Research Centre, The Institute of Cancer Research, London, UK; 5 Cancer Biomarkers & Biotherapeutics, Barts Cancer Institute, Queen Mary University of London, John Vane Science Building, London, UK; 6 Medical Oncology, National Center for Tumor Diseases, University Hospital Heidelberg, Heidelberg, Germany; 7 Else Kroener Fresenius Center for Digital Health, Medical Faculty Carl Gustav Carus, TUD Dresden University of Technology, Dresden, Germany

**Keywords:** Cancer imaging, Predictive markers, Tumour biomarkers

## Abstract

**Background:**

The objective of this comprehensive pan-cancer study is to evaluate the potential of deep learning (DL) for molecular profiling of multi-omic biomarkers directly from hematoxylin and eosin (H&E)-stained whole slide images.

**Methods:**

A total of 12,093 DL models predicting 4031 multi-omic biomarkers across 32 cancer types were trained and validated. The study included a broad range of genetic, transcriptomic, and proteomic biomarkers, as well as established prognostic markers, molecular subtypes, and clinical outcomes.

**Results:**

Here we show that 50% of the models achieve an area under the curve (AUC) of 0.644 or higher. The observed AUC for 25% of the models is at least 0.719 and exceeds 0.834 for the top 5%. Molecular profiling with image-based histomorphological features is generally considered feasible for most of the investigated biomarkers and across different cancer types. The performance appears to be independent of tumor purity, sample size, and class ratio (prevalence), suggesting a degree of inherent predictability in histomorphology.

**Conclusions:**

The results demonstrate that DL holds promise to predict a wide range of biomarkers across the omics spectrum using only H&E-stained histological slides of solid tumors. This paves the way for accelerating diagnosis and developing more precise treatments for cancer patients.

## Plain language summary

Molecular profiling tests are used to check cancers for changes in certain genes, proteins, or other molecules. Results of such tests can be used to identify the most effective treatment for cancer patients. Faster and more accessible alternatives to standard tests are needed to improve cancer care. This study investigates whether deep learning (DL), a series of advanced computer techniques, can perform molecular profiling directly from routinely-collected images of tumor specimens used for diagnostic purposes. Over 12,000 DL models were utilized to evaluate thousands of biomarkers using statistical approaches. The results indicate that DL can effectively detect molecular changes in a tumor from these images, for many biomarkers and tumor types. The study shows that DL-based molecular profiling from images is possible. Introducing this type of approach into routine clinical workflows could potentially accelerate treatment decisions and improve outcomes.

## Abstract (web summary)

Arslan et al. present the results of a comprehensive pan-cancer study evaluating deep learning-based multi-omic biomarker profiling using H&E-stained whole slide images. They show that deep learning can predict a wide range of biomarkers across the omics spectrum and in different cancers directly from histomorphology.

## Introduction

Studying the alterations at different levels of the molecular landscape helps better understand oncogenesis and cancer progression^1,2^. In-depth analysis of the associations between the molecular aberrations and the tumor microenvironment has enabled the development of targeted therapies in various cancer types^3–5^. Genetic profiling has become an important tool^6^, especially for individuals who possess a higher risk of developing cancer due to genetic factors^7^. There is also an increasing need for alternative solutions to standard molecular and genomic profiling methods, which are often accountable for laboratory delays in the routine clinical workflow, as they take time to prepare, process, and analyze^8,9^. In addition, expensive tests may not be routinely accessible to all patients^10^.

Meanwhile, there has been accumulating evidence suggesting that diagnostic histology images stained with hematoxylin and eosin (H&E) may contain information that can be used to infer molecular profiles directly from histological slides^11,12^. Deep learning (DL) can effectively reveal differences in morphological phenotypes in malignancies, which in turn, enables the prediction of molecular profiles directly from H&E-stained whole slide images (WSIs)^11–15^. DL-based methods have been used to infer molecular alterations in various cancer types, including breast^9,16–18^, colorectal^13,15,19,20^, lung^12,21^, gastric^22^, prostate^23^, skin^24^, and thyroid^25^ (see Echle et al. for an extensive review of DL applications for biomarker profiling from histology images^26^). More recently, pan-cancer studies have explored the links between genetic/molecular alterations and histomorphological features in H&E images. These studies showed that in almost all malignancies, DL methods can be used to infer a plethora of biomarkers directly from routine histology^11,14,15,27–29^.

Expanding upon previous work, we conducted a large-scale study to assess the feasibility of biomarker profiling from routine diagnostic slides with DL. The investigated biomarkers included a broad range of genomic, transcriptomic, and proteomic alterations as well as various downstream biomarkers with clinical relevance (e.g., standard of care features, molecular subtypes, gene expressions, clinical outcomes, and response to treatment). We systematically evaluated predictability across all solid cancers studied by the Cancer Genome Atlas (TCGA) program. Our DL approach utilizes an autoencoder network as a feature extractor that enables learning representations of the histology images that are relevant to the profiling task at hand. In contrast to previous studies, we have extended the scope of assessing pan-cancer predictability with DL to a wider range of cancer types (*n* = 32) and to thousands of biomarkers across the central dogma of molecular biology (*n* = 4031). The systematic predictability of many of these biomarkers from routine histology has not been assessed at a large scale before, including certain phenotypic and clinical outcomes such as drug responses.

Our findings suggest that multi-omic biomarkers can potentially be predicted directly from histomorphology. Profiling mutations from histology is mostly feasible for the majority of genes tested. Frequently mutated genes like *TP53* are predictable across multiple cancer types. It is possible to predict the under-/over-expression status of transcriptomes and proteins to a certain degree. The morphological visual characteristics can be detected with DL, enabling the prediction of molecular subtypes and well-established clinical biomarkers directly from WSIs. Similar results are acquired when our experiments are repeated with an external dataset based on certain cancer types and biomarkers that have equivalents in the TCGA dataset, further confirming the general feasibility of predicting pan-cancer biomarkers from H&E-stained slides. Considering various factors that may have an impact on predictability, such as tumor purity, sample size, and prevalence of biomarker status, we conclude that there potentially exists a degree of true predictability that may be associated with histomorphology.

## Methods

### Dataset

We conducted our experiments on the data provided by TCGA project, which was retrieved via the Genomic Data Commons (GDC) Portal (https://portal.gdc.cancer.gov/). The TCGA dataset consisted of 10,954 hematoxylin and eosin (H&E)-stained, formalin-fixed, and paraffin-embedded (FFPE) whole slides images of 8890 patients, acquired from the following studies: breast cancer (BRCA), cervical squamous cell carcinoma, kidney renal papillary cell carcinoma (KIRP), clear cell renal cell carcinoma (KIRC), kidney chromophobe (KICH), skin cutaneous melanoma, sarcoma (SARC), pancreatic adenocarcinoma, ovarian serous cystadenocarcinoma (OV), prostate adenocarcinoma, bladder urothelial carcinoma, esophageal carcinoma (ESCA), thyroid carcinoma (THCA), lymphoid neoplasm diffuse large B-cell Lymphoma (DLBC), brain lower-grade glioma (LGG), thymoma (THYM), head and neck squamous cell carcinoma (HNSC), uterine corpus endometrial carcinoma (UCEC), glioblastoma multiforme (GBM), cholangiocarcinoma, liver hepatocellular carcinoma, stomach adenocarcinoma, lung adenocarcinoma (LUAD), and lung squamous cell carcinoma (LUSC), colon adenocarcinoma (COAD), rectum adenocarcinoma, adrenocortical carcinoma (ACC), mesothelioma (MESO), pheochromocytoma and paraganglioma, testicular germ cell tumors, uterine carcinosarcoma (UCS), and uveal melanoma (UVM). Only images scanned at a resolution of 0.5 microns per pixel (MPP) were kept and images with no MPP information were discarded, ensuring consistent resolution within each cancer cohort. The number of the images and patients included in the TCGA cohort are provided in Supplementary Table 1. DLBC, UVM, and THYM were excluded from certain results due to having less than seven valid targets considering all biomarker types. See the Biomarker acquisition section for more details on the biomarker inclusion criteria for each omic/biomarker type.

### External dataset

In order to assess the general feasibility of biomarker predictability on an external dataset, we repeated our experiments with the Clinical Proteomic Tumor Analysis Consortium (CPTAC) data, retrieved via the Cancer Imaging Archive (https://wiki.cancerimagingarchive.net/display/Public/CPTAC+Imaging+Proteomics). The CPTAC dataset consisted of 3481 H&E stained images corresponding to 1329 patients, acquired from the following seven different studies: LUAD, COAD, head-and-neck cancer (HNSCC), LSCC, pancreatic ductal adenocarcinoma (PDA), GBM, and UCEC. LUAD, GBM, and COAD were obtained from frozen tissues, whereas the rest of the datasets contained FFPE slides. Images with a resolution different than 0.25 MPP in COAD and 0.5 MPP in other datasets were discarded, ensuring consistent resolution within each cancer cohort. The details of the final images and patients included in the CPTAC cohort are provided in Supplementary Table 2.

### Biomarker acquisition

#### Acquisition of actionable driver genes

Clinically relevant driver genes were retrieved from https://cancervariants.org^30^. We only considered driver genes that are known to associate with (1) Food and Drug Administration (FDA)-approved disease-specific therapies and (2) response or resistance to therapies as shown in professional guidelines and/or based on well-powered studies with consensus from experts in the field based on evidence provided in another study^31^. Driver mutation and drug-associated data were acquired from the following sources: BRCA exchange, the Cancer Genome Interpreter Cancer Biomarkers Database, Clinical Interpretation of Variants in Cancer, Jackson Laboratory Clinical Knowledgebase (JAX-CKB), the Precision Medicine Knowledgebase. These source files already contained the associations between SNP mutations and phenotypes, allowing an expert pathologist to map them to TCGA studies. Finally using this mapping and driver mutation data per phenotype, we created a set of driver genes per TCGA study and subsequently used them to filter actionable biomarkers for transcriptomic, proteomic, and genomic data.

#### TCGA genomic biomarker profiles

Genomic biomarker data was collected using the cBioPortal web API and the GDC API. For each TCGA study, we retrieved all samples with associated diagnostic slides. The samples that did not have whole-genome or whole-exome sequencing data were excluded from further consideration, allowing us to assume that all genes of interest were profiled across the remaining ones. While there existed samples without WGS or WXS data with mutations, it was not possible to assume that genes with no mutations were present in their wild-type, as they might simply have not been sequenced. For all TCGA studies listed on the cBio portal, we acquired molecular profiles with the “MUTATION_EXTENDED” alteration type and all mutations belonging to these molecular profiles within the collected samples were retrieved and stored in an intermediate format. Finally, we created molecular profiles for all driver genes using this mutation data. A sample was considered positive for a driver gene if it contained at least one single nucleotide variant (SNV). SNVs are typically insertions, replacements, or deletions of one base, but on a few occasions can be of multiple bases (e.g., “T” is replaced by “CGC”). The resulting profiles were filtered to exclude driver genes that had less than ten positive samples in a given cancer.

#### Transcriptomic and proteomic profiles

Transcriptomic and proteomic data for TCGA datasets were retrieved from the cBioPortal API. cBioPortal provides z-scores, which were originally computed from the raw FPKM counts of gene expression and the corresponding number of standard deviations to the mean of expression values. These z-scores were acquired for each coding gene and each sample with an associated tissue slide in the TCGA studies. cBioPortal restricted the transcriptome z-score calculations to the samples in which the tumor comprised diploid cells. Proteomic z-scores were calculated among all available samples for a given cancer. The z-scores were binarised for each gene and sample based on thresholds chosen as follows: For each sample, genes with a z-score of less than or equal to t_under were considered underexpressed and those with a z-score of larger than or equal to t_over were considered overexpressed. We set {t_under, t_over} to {−2, 2} and {−1.5, 1.5}, for transcriptomic and proteomic data, respectively, based on their ability to divide the total distribution of z-scores into balanced classes over all genes of interest. These thresholds were then used to generate two under-/over-expression profiles for proteomic and transcriptomic genes. In an under-expression profile all samples that were considered underexpressed were labeled as positive whereas all other samples were labeled as negative. Similarly, overexpressed samples in an over-expression profile were assigned a positive label, while the remaining samples were considered negative. Finally, to reduce the number of target biomarkers, we limited the over-and under-expression profiles to only include the driver genes (see Acquisition of actionable driver genes) for each study. Furthermore, profiles that did not contain enough positive samples were excluded. The minimum number of positive samples for proteomic genes was set to 20. For the transcriptomic profiles, only the ones with at least a positive ratio of 10% and having a minimum of ten positive samples were kept.

#### Standard of care features, gene expression signatures, and molecular subtypes

A publicly available dataset provided as part of a relevant study on detecting clinically actionable molecular alterations was used to acquire the biomarker profiles for gene expression signatures, molecular subtypes, and standard clinical biomarkers^11^. The dataset was originally curated from the results of systematic studies using the TCGA data (https://portal.gdc.cancer.gov/)^32–34^ and contained profiles for 17 TCGA datasets (please refer to the original study^11^ for the description of biomarkers and other details regarding the acquisition protocol). For certain biomarkers, we used the consensus opinion to map the molecular status to binary labels. For instance, considering microsatellite instability (MSI), all patients defined as MSI-H were included in the positive class, while microsatellite stable (MSS) and MSI-L patients were labeled as negative. Profiles with multiple categorical values were binarised with one-hot-encoding, where a profile was created for each category with only the samples of that category being set to positive. Non-categorical profiles with continuous values were binarised at mean after eliminating NaN values^11^.

#### Clinical outcomes and treatment responses

Survival data was acquired from the TCGA Pan-Cancer Clinical Data Resource (TCGA-CDR), a publicly available dataset that provides four major clinical outcome endpoints^35^, namely, overall survival (OS), disease-specific-survival (DSS), disease-free-interval (DFI), and progression-free interval (PFI). These endpoints were systematically binarized into actionable events by considering multiple clinical and prognostic features acquired from TCGA’s routinely-collected clinical data (https://portal.gdc.cancer.gov/) such as vital status, tumor status, cause of death, new tumor events, local recurrence and distract metastasis. The details of the integration of the clinical data into actionable survival outcomes are given in the original study^35^ and summarized in Supplementary Table 3. Additionally, we added the residual tumor status acquired from the TCGA clinical files as another prognostic target. Patients with microscopic or macroscopic residual tumors (R1 or R2) were classified as positive whereas those with no residual tumor (R0) were included in the negative class^36^. Since TCGA-BRCA did not have residual tumor information, we used the “margin_status” attribute. Similarly, “treatment_outcome_first_course” was used to create binary targets representing the treatment response. Towards this end, any patient with “Complete Remission/Response” was included in the positive class whereas “Stable Disease”, “Partial Response” and “Progressive Disease” were considered negative. Finally, clinical drug files in the TCGA datasets were used to identify drug responses. This was achieved by first unifying drug names based on the data provided in another study^37^ and then identifying drug-study pairs with enough samples. Finally, the “treatment_best_response” attribute was used to map the drug responses to binary categories, with “ Complete Response” constituting the positive class and the others being negative. For both treatment and drug responses, we only focus on assessing the predictability of complete response from histology and classify other outcomes including partial response in the negative class.

#### CPTAC genomic biomarker profiles

Molecular profiling data was collected using the GDC API. We focused on the “Single Nucleotide Variation” (SNV) files, which contained information about mutations associated with substitutions of a single DNA base or deletion and insertion of a small number of bases. The SNV files were retrieved using the files endpoint of the GDC API, with the following filters: files.data_category = [“Simple Nucleotide Variation”], files.data_type = [“Masked Somatic Mutation”] and files.experimental_strategy = [“WXS”]. After acquiring the SNV files, it was possible to obtain a list of SNV gene mutations for each sample of the CPTAC dataset. Since the mutation data was based on whole exome sequencing, a gene was considered wild-type if it was not listed in the SNV file of a given sample. Finally, biomarker profiles were limited to only include the driver genes (see Acquisition of actionable driver genes). A sample was considered positive if the sample contained at least one mutation for a given driver gene. The resulting profiles were filtered to exclude genes that had less than 10 positive samples in a given dataset.

### Experimental setup

We assessed the predictive performance of each biomarker in a 3-fold cross-validation setting, where the cases with a valid biomarker status in each dataset were split into three random partitions (folds), each having approximately the same proportion of positive samples. We trained and tested three models per biomarker, each time keeping aside a different fold for validation and using the remaining ones for training. This setting ensured that the predictability can be assessed on a different (hold-out) validation set for multiple times, and consequently allowing us to assess the variability of model performance. The images were partitioned at the patient level so that no patient could appear in multiple folds. A biomarker profile with less than ten positive patients was discarded from the study.

### Pre-processing pipeline and training details

**Fig. 1:**
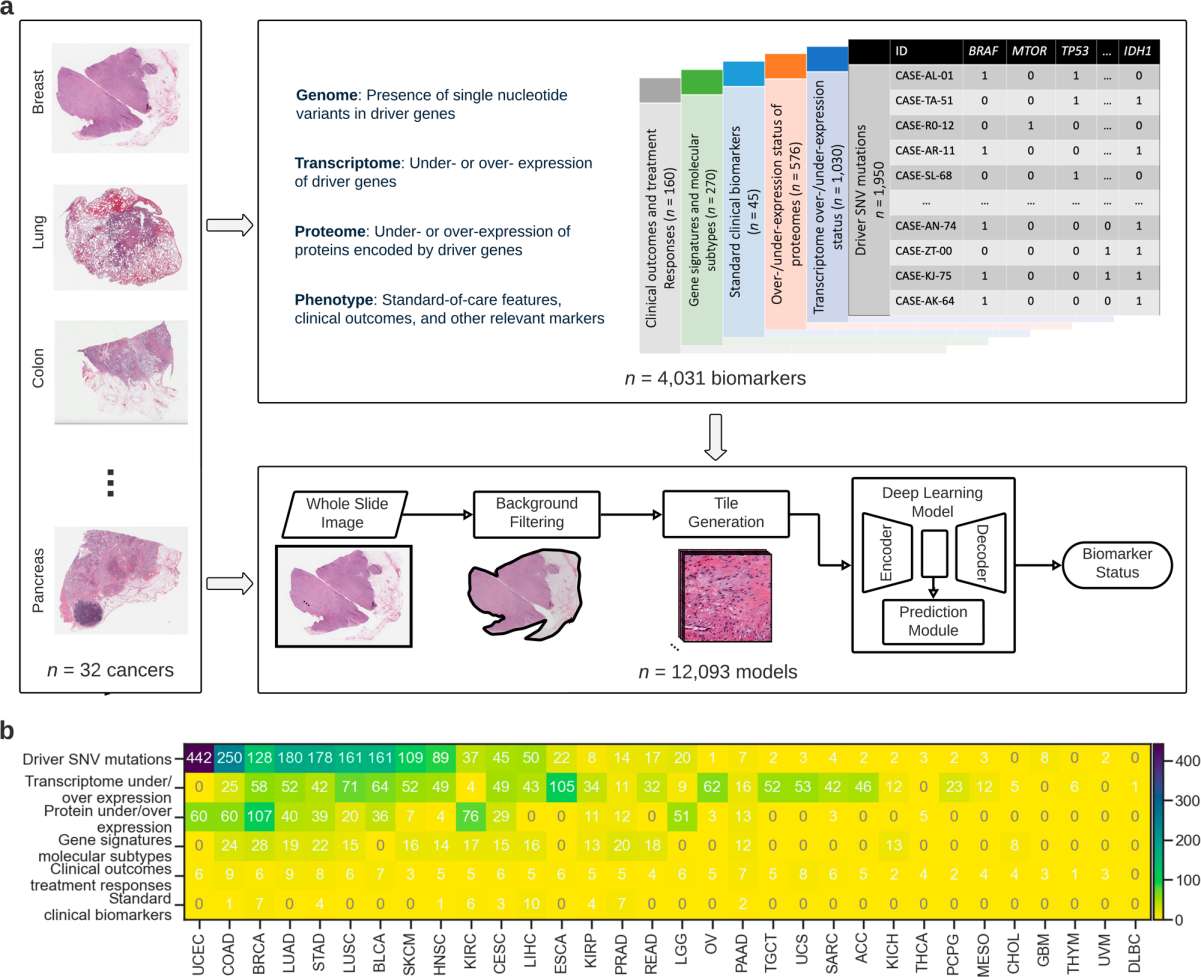
Deep learning for molecular profiling from routine histology images. **a** Overview of the pre-processing and training pipeline used for assessing the feasibility of predicting a plethora of genomic, transcriptomic, and proteomic biomarkers as well as various clinically-relevant biomarkers (e.g., standard of care features, molecular subtypes, clinical outcomes, response to treatment) with deep learning from whole slide images stained with hematoxylin and eosin (H&E). A convolutional neural network (CNN) consisting of an encoder (i.e., feature extractor), a decoder, and a classification module was used for predicting molecular profiles from H&E images (see Methods: Pre-processing pipeline and training details). A single CNN model was end-to-end trained from scratch for each biomarker. Each slide was parcellated into a set of 256×256 tiles and those that did not contain any tissue were automatically discarded. The remaining tiles were assigned with a ground-truth molecular profile (see Methods: Biomarker acquisition). **b** The number of biomarkers per cancer type is shown as a heatmap. Biomarkers are grouped according to omics category and cancer type. SNV refers to single nucleotide variant. Cancer abbreviations are defined in Supplementary Table 1.

A convolutional neural network (CNN) was used for predicting molecular profiles from H&E images as illustrated in Fig. 1. A single CNN was end-to-end trained from scratch for each biomarker and fold, yielding a total of 12,093 unique models that were used to obtain the results presented in this study. Each model was trained on a set of 256 × 256 tiles acquired from H&E-stained WSIs, considering the whole histological material. A standard deviation filter was used to eliminate the tiles which do not contain any relevant information, allowing us to extract the tissue from the rest of the image. A slide was discarded from analysis if it contained fewer than ten tiles after the filtering process. Macenko color and brightness normalization^38^ was applied to the remaining tiles before they were assigned with a ground-truth molecular profile (see Biomarker acquisition).

A CNN consisted of a feature extractor (encoder), a decoder and a classification module. The encoder can capture the tissue properties within tiles throughout a set of convolutional filters applied to tiles at various layers of depth, effectively encoding the high-level visual features into a d-dimensional feature vector, where d depends on the architecture of the CNN. These vectors are regarded as the fingerprints of the tiles and are submitted to both the decoder and the classification module. The decoder module takes a d-dimensional embedding as input and returns an output of the same shape as the original tile that the embedding represents. It consists of a series of transposed convolutional and upsampling layers that is used to reconstruct the original tile from the latent vector to achieve better representations of each tile that do not contain irrelevant features. The output of the decoder is compared against the original tile with mean squared error (MSE), or reconstruction loss. In the meantime, the output of the encoder, i.e., the d-dimensional feature vector, is submitted to the classification module, which consists of a fully-connected dense layer with softmax nonlinearity and performs the actual classification task. The output of this module, i.e., classification probability, is then compared to the ground-truth label associated with the WSI and a cross-entropy (CE) loss is produced. CE-loss is finally added to the MSE loss to acquire the total loss. By back-propagating on this combined loss function, we train the model to output classification scores that are closer to the tile-level targets while achieving representations of each tile that are independent of image noise (i.e., irrelevant features).

Model hyperparameters and the CNN architecture were determined based on a relevant benchmark analysis from the clinical validation study of a DL model developed for molecular profiling of BRCA^39^. We adopted the best-performing model’s feature extractor network (based on a “resnet34” architecture^40^) and hyper-parameters to configure a CNN for each biomarker in the current pan-cancer study. Our model selection is further endorsed by a recent benchmarking study comparing weakly-supervised DL approaches for biomarker profiling in computational pathology, where the tile-based DL models are shown to likely outperform newer architectures, such as those based on multi-instance learning, in classification tasks^41^. Each model was trained for 10 epochs using the Adam optimizer with a learning rate of 0.0001. A total of 200 tiles were randomly sampled from each of the training slides and oversampling was applied to the tiles from the underrepresented class to ensure that there is roughly a 50–50 representation of each class during training. During validation, predictions across all tiles were averaged to determine a slide-level prediction. Validation AUC was monitored as the target metric to select the final model during training. Classification scores across images of the same patients were averaged to compute AUC values at patient level.

### Tumor purity experiment

#### TCGA tumor purity data

We obtained the tumor composition data from the biospecimen slide files named as “nationwidechildrens.org_biospecimen_slide_${study_name}.txt”. These files are part of the TCGA metadata and can be downloaded from https://portal.gdc.cancer.gov/. We extracted the “percent_tumor_cells” property that measures the proportion of the tumor cells in a tissue image and used it as an estimate for tumor purity. For cases where multiple tissue slides were available, a single tumor purity value was calculated by averaging the corresponding “percent_tumor_cells” measurements. Cases lacking this property were excluded from the tumor purity experiment.

#### Experimental details

For assessing the capability of tumor purity to predict the endpoints (Supplementary Fig. 1), we employed the experimental setup used for H&E-driven biomarker profiling. However, instead of predicting on image-based features (*X*_*H*&*E*_), we opted to use tumor purity (*X*_*TP*_) to determine the biomarker status (*y*). Tumor purity was defined as the percentage of tumor cells in a tissue slide, which is provided for each case in the TCGA metadata (refer to TCGA tumor purity data for details). Using the same 3-fold cross-validation setting (see Experimental setup), we trained and tested three random forest (*f*_*RF*_) classifiers per biomarker, each time reserving a different fold for validation and using the remaining folds for training. In total, 12,093 *f*_*RF*_ classifiers were trained across 4031 distinct endpoints (biomarkers) and 32 cancer types, with the classification performance being evaluated using the AUC metric.

Following the same experimental setup established a one-to-one correspondence between image-driven DL models (*f*_*DL*_*)* and classifiers running on tumor purity (*f*_*RF*_*)* for all studied biomarkers. This facilitated a direct comparison of the prediction tasks defined as ƒ_DL_(X_H&E_) = y and ƒ_RF_(X_TP_) = y, where *X*_*TP*_ *and X*_*H*&*E*_ denote the inputs to the classifiers and *y* corresponds to the biomarker status. The significance of the difference in performance between the *f*_*DL*_ and *f*_*RF*_ classifiers was assessed using a two-sided t-test (see Performance characteristics and statistical Procedures for details**)**. This comparison was conducted separately for each subgroup of biomarkers to provide increased granularity.

Additionally, we explored the linear relationship between tumor purity and predictability, as indicated by model performance. The percentage of tumor cells used to estimate tumor purity was averaged within the samples of each biomarker and compared with the AUCs of the DL models using the Pearson correlation coefficient (PCC). This correlation analysis was performed separately for each subgroup of biomarkers.

#### Performance characteristics and statistical procedures

Performance of a model was measured with the area under receiver operating characteristic curve (AUC), which plots the relationship between True Positive Rate and False Positive Rate across different predictive thresholds. An AUC of 0.5 denotes a random model, while a perfect model that can predict all samples correctly yields an AUC of 1. For each biomarker, we reported the performance as the average AUC across the three models (unless otherwise specified), accompanied with the standard deviation (denoted with ±where appropriate).

Statistical significance of results were determined with a two-sided t-test using the “ttest_ind” function from the python scipy-stats library. This is a test for the null hypothesis and assumes that two independent samples have identical expected values and variances. For testing the significance at the group level, the measured AUC values were compared against a randomly-sampled set of values with the same underlying variance. For instance, to measure the statistical significance of the difference between the AUC values acquired for a specific cancer and biomarker type, all AUC values from that group were compared against a set of random AUC values sampled from a distribution with a mean of 0.5 (resembling random performance) and the standard deviation of the group in comparison. To determine the statistical significance of the predictability at biomarker level, we applied the same test on the prediction scores obtained from the true negative and positive cases. Towards this end, classification scores from all three folds of a biomarker were retrospectively combined and the scores from the positive cases and those from the negative class were compared with a two-sided t-test to determine if the difference between the negative and positive predictions were statistically significant. The resulting *p*-values were corrected for multiple testing using the Benjamini-Hochberg procedure with a false discovery rate (FDR) of 0.05. All biomarkers with an adjusted p-value of less than 0.05 were considered statistically significant.

#### Ethics oversight

Only retrospective and publicly-available data was used. The authors had no role in the recruitment of participants. Therefore, no ethical approval is required.

### Reporting summary

Further information on research design is available in the Nature Portfolio Reporting Summary linked to this article.

## Results

### DL for molecular profiling from routine histology images

A CNN was used for predicting molecular profiles from H&E images as illustrated in Fig. 1a and explained in Methods. Our CNN consisted of an encoder (i.e., feature extractor), a decoder, and a profiling module for classification. In conventional CNNs, morphological features acquired from histology images are directly correlated with the target molecular profile. In our approach, the combined classification and encoder-decoder architecture enable learning a better representation (i.e., encoding) of morphological images that are free of irrelevant features like image noise. The investigated biomarkers in our study included genetic alterations in driver genes, over-and under-expression of driver genes and relevant proteins, established biomarkers that are routinely used in clinical management, clinical outcomes such as OS and treatment responses, biomarkers that are highly relevant for prognosis and targeted therapies, including molecular subtypes and gene expression signatures (Methods: Biomarker acquisition). We assessed the predictive performance of each biomarker in a 3-fold cross-validation setting, where the cases with a valid biomarker status in each cohort were split into three random partitions (folds), each having similar proportions of positive/negative samples. We trained and tested three models per biomarker, each time keeping aside a different fold for testing and using the remaining ones for training. This setting ensured that a test prediction could be acquired for each patient and allowed us to assess the variance in biomarker performance. The predictability of a biomarker was measured with the area under the receiver operating characteristic curve (AUC). For each biomarker, we reported the performance as the average AUC across the three models (unless otherwise specified), accompanied by the standard deviation (denoted with ±where appropriate) measuring intra-marker variability. In total, 12,093 models were trained across 4031 distinct biomarkers and 32 cancer types, with the following breakdown (Fig. 1b): 1950 SNV mutations in driver genes, 1030 transcriptome expression level markers, 576 protein expression level markers, 270 gene signature and molecular subtype markers, 160 markers related to clinical outcomes and treatment responses and 45 standard-of-care markers.

### Pan-cancer predictability of multi-omic biomarkers from routine histology images with DL

**Fig. 2:**
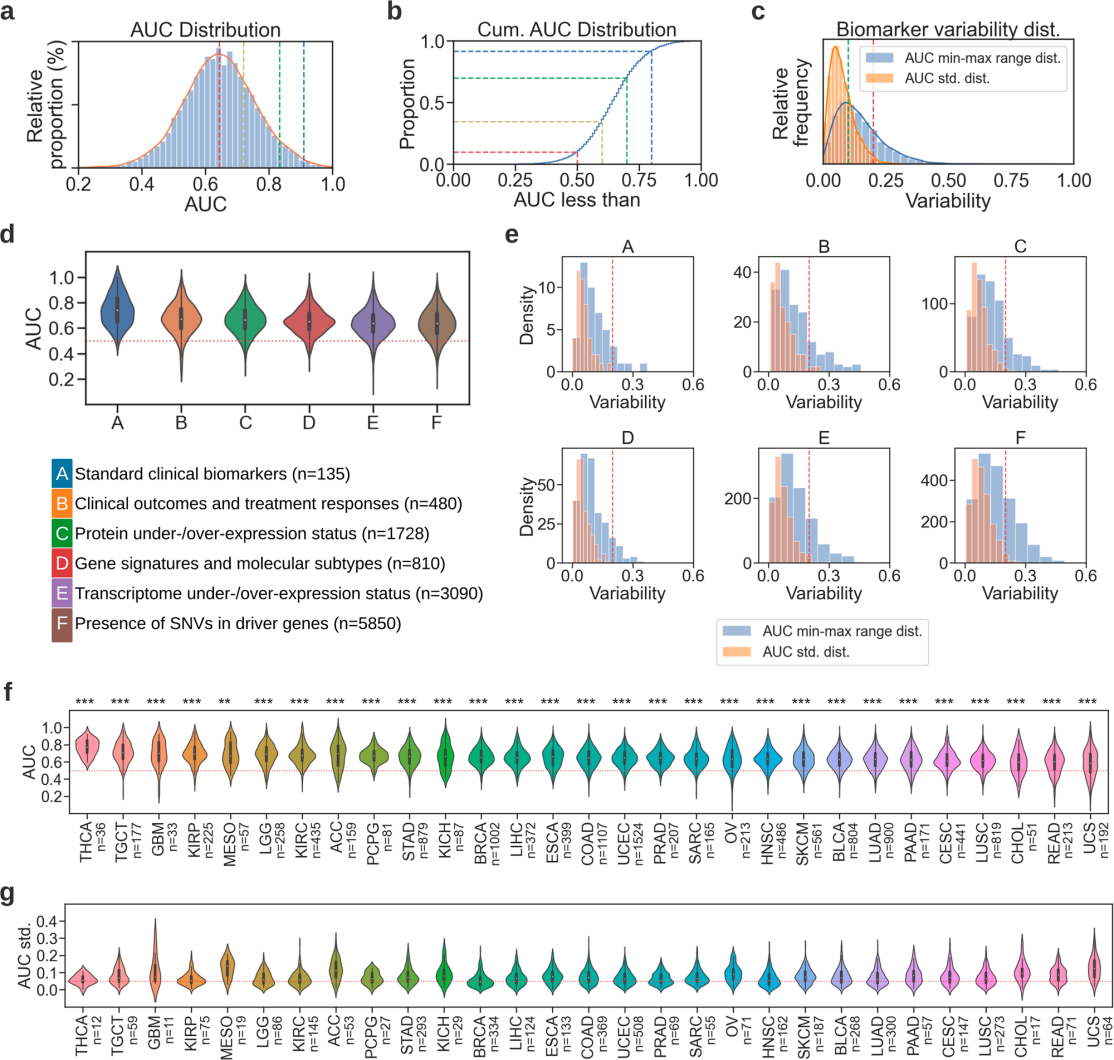
Multi-omic biomarkers can be predicted directly from histomorphology across multiple cancer types. **a** Histogram distribution and kernel density estimation of the area under the curve (AUC) values for all models (*n* = 12,093), where the markers indicate the proportion of models at 50%, 75%, 95%, and 99%. **b** Cumulative AUC distribution shows the proportion of models that have AUC less than the shown markers at 0.5, 0.6, 0.7, and 0.8. **c** Standard deviation and min-max range distribution of model performance in AUC. **d** Violin plots showing the AUC distribution of each biomarker type. The same coding in Table 1 was used to abbreviate the biomarker types. Box plots were used to represent the data points in violins, with whiskers showing the 1.5x interquartile range and median values indicated with white dots. **e** Standard deviation (orange histogram) and min-max range distribution (blue histogram) of model performance in AUC across the cross-validation folds. **f** Violin plots showing the AUC distribution per cancer type (see Supplementary Table 1 for cancer abbreviations). Plots are sorted by average intra-study AUC. Lymphoid neoplasm diffuse large B-cell Lymphoma (DLBC), uveal melanoma (UVM), and thymoma (THYM) were excluded from this analysis due to only constituting one to seven valid targets across all biomarker types. The number of models per cancer type is given in parentheses. **g** Violin plots showing the standard deviation distribution of model performance across different folds of each biomarker, in the same order as in (**f**). The number of standard deviation values per cancer type is given in parentheses.

We assessed the overall feasibility of profiling biologically different biomarkers using histomorphological characteristics of standard H&E-stained WSIs. Half of the models achieved an AUC of 0.644 or higher. The observed AUC was greater than or equal to 0.719 for 25% of the models and above 0.834 for 5%. The top 1% of models (*n* = 122) returned an AUC of at least 0.909 (Fig. 2a, b). For the majority of the biomarkers we investigated, the predictability was mostly consistent, with the standard deviation being less than 0.1 AUC and the difference between the minimum and maximum performance being less than 0.2 AUC (Fig. 2c). The majority of the biomarkers under investigation showed better-than-random performance across all omics/biomarker types (Fig. 2d, Table 1). The lowest average performance was seen in the prediction of SNVs in driver genes (AUC 0.636 ± 0.117), and the highest performing models were from the standard clinical biomarkers (AUC 0.742 ± 0.120). Variability across different models of the same biomarker type showed similar trends compared to that in the overall distribution, with the standard deviation for all omics being around 0.2 AUC and an increase in variance considering the range in minimum-maximum performance (Fig. 2e).

**Table 1:** Average performance and standard deviation for all omic types, alongside their brief description

Biomarker type/omic	Description	Mean AUC ± std.
Standard clinical biomarkers	Established biomarkers that are routinely used in clinical management, as described in a related study^11^.	0.742 ± 0.120 (*n* = 135)
Clinical outcomes and treatment responses	Clinical outcomes such as overall survival and treatment responses to therapy or drugs.	0.671 ± 0.120 (*n* = 480)
Under-/over-expression of proteins	Under- and/or over-expression status of proteins encoded by driver genes.	0.666 ± 0.108 (*n* = 1728)
Gene signatures and molecular subtypes	Biomarkers that are highly relevant for prognosis and targeted therapies, including molecular subtypes and gene expression signatures, as compiled in a related study^11^.	0.653 ± 0.097 (*n* = 810)
Under-/over-expression of transcriptomes	Under- and/or over-expression status in driver genes at the transcript level.	0.637 ± 0.108 (*n* = 3090)
Presence of single nucleotide variants (SNVs)	The presence of SNVs in driver genes, associated with FDA-approved therapies or known-relevance for specific treatments.	0.636 ± 0.117 (*n* = 5850)

The table is sorted by the average performance in descending order. The number of biomarkers tested for each biomarker type is given in parentheses in the last column.

To show a more granular overview of the biomarker performance across cancers, we plotted the distribution of AUC values for all the investigated malignancies with sufficient samples (Fig. 2f) and provided the average performance with standard deviations in Supplementary Table 4. Overall, performance was significantly better than random across all cancer types (i.e., mean AUC > 0.5 and *p* < 1e–05 for all malignancies, where statistical significance for each cancer was measured with a two-sided t-test performed between the AUC values of all models belonging to a cancer type and a set of random AUC values with a mean of 0.5 and the same standard deviation as the compared AUC distribution). The lowest general performance was obtained in UCS with a mean AUC of 0.585 (±0.158), and the highest performing models were in THCA, where an average AUC of 0.768 (±0.091) was measured. The variability across cross-validation folds of each biomarker was mostly stable, with standard deviations centering around 0.05 AUC in most of the studies (Fig. 2g). A breakdown of the overall predictability performance for each malignancy depending on the type of biomarker is provided in Supplementary Fig. 2.

### Feasibility of predicting genetic alterations from histology

Recent pan-cancer studies have shown that mutations can be detected from histomorphological features with DL^11,14^. In our study, we extend the previous work on predicting mutational status to a total of 1950 genomic biomarkers. We focused on predicting the SNVs in driver genes that are associated with disease-specific therapies approved by the FDA or are known to be relevant for specific treatments based on evidence from clinical guidelines or well-powered studies with consensus from experts in the field^31^. In our experiments, we used the genomic profiles available from the TCGA project.

**Fig. 3:**
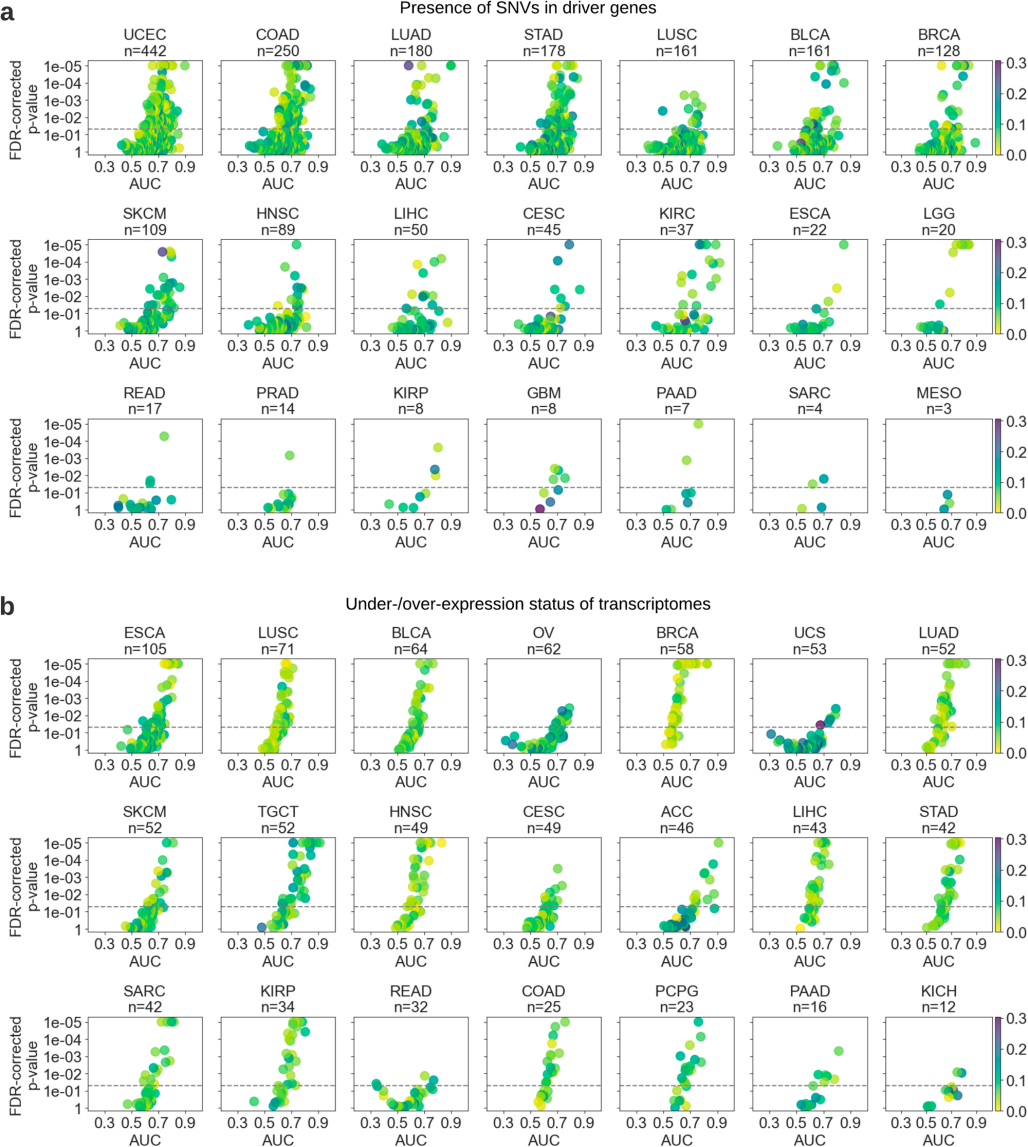
Deep learning could predict genetic alterations and transcriptome expression status from routine histology images across many cancer types. Scatter plots show the average test area under the curve (AUC) for each model trained to predict (**a**) presence of single nucleotide variants (SNVs) in driver genes and (**b**) under-/over-expression status of transcriptomes across selected cancer types. Each marker in the scatter plot represents a tested biomarker. A two-sided *t*-test was applied to prediction scores of each model to assess the statistical significance and the corresponding *p*-values were corrected for false discovery rate (FDR). The y-axis of each plot was inverted and the *p*-values were log-transformed for visualization purposes. *p* values smaller than 1e–05 were set to 1e–05 to avoid numerical errors during transformation. The statistical significance threshold of 0.05 is marked with a dashed line. Color shading of the markers indicates the standard deviation of the predictability performance for each biomarker. Plots are ordered by the number of tested biomarkers in each row. Due to space limitations, cancer types with few biomarkers are not shown in this figure but are provided in Supplementary Fig. 9. Cancer abbreviations are defined in Supplementary Table 1.

Genetic alterations were significantly predictable across most of the investigated cancer types (Fig. 3a), with a mean AUC of 0.636 (±0.117). More than 40% of the mutations were detectable with an AUC of at least 0.65, and considering the highest performing mutations in each cancer type, almost all major malignancies had at least ten genes with mutations being predictable at an AUC level of 0.70 or above. Among them, endometrial carcinoma had the highest number of predictable mutational status (*n* = 112 out of 442 genes). It was followed by colon cancer (*n* = 62 out of 250 genes), gastric cancer (*n* = 58 out of 178 genes), skin melanoma (*n* = 29 out of 109 genes), LUAD (*n* = 28 out of 180 genes), and BRCA (*n* = 26 out of 128 genes). Among all the tested genes, the top-performing ones were *NUMA1* and *JAK1* in KIRC, *PDGFRB* and *BCL6* in lung cancer, *IRS2* in endometrial cancer, and *GNAS* in BRCA, each with an AUC of at least 0.89. A large number of genes were highly predictable across multiple cancer types (Supplementary Fig. 3a). Notably, SNV alterations in *TP53* were detectable in many malignancies, with 7 out of 22 cancers tested having an AUC of at least 0.7 and 14 of them showing AUCs greater than 0.65, reaching up to 0.841 for brain LGG, up to 0.785 for BRCA and up to 0.771 for endometrial cancer.

### Feasibility of inferring under-/over-expression of transcriptomes from diagnostic histology slides

Analysis of gene expressions is fundamental to better understanding the underlying cancer mechanisms and holds promise in improving cancer diagnosis and facilitating drug discovery^42^. While it is already known that genetic alterations could potentially be detected from histomorphology with DL, studies to understand the extent of predictability at transcript and protein levels have been rather limited. Recently, it has been shown that transcriptomic profiles are correlated with histomorphological features detected with DL in an annotation-free setup^15^. Our study took a more direct and comprehensive approach and trained DL models to predict the under- and/or over-expression status in selected driver genes, using the transcriptomic profiles available from TCGA. We identified 97 genes qualified for studying the predictability of under-expression and 933 genes qualified for studying the predictability of over-expression at the transcript level across different cancers.

The over-/under-expression status for the investigated genes was mostly predictable across the majority of cancer types (Fig. 3b) with a mean AUC of 0.637 (±0.108). The average performance was slightly lower for the under-expressed genes (mean AUC 0.633 ± 0.115). Expression status in at least 40% of the genes was detectable with an AUC of 0.65 or above. ESCA and testis cancer were the malignancies with the highest number of genes with a predictable expression status (a total of 28 out of 105 and 52 genes, respectively), defined as an AUC level of at least 0.70. It was followed by ovarian cancer (18 out of 62 genes) and ACC (16 out of 46 genes). Almost all of the top-performing genes were that of over-expression, with *PMS2* in THYM; *CARD11*, *LASP1*, *STIL, POLE, KMT2C*, and *CLIP1* in testis cancer; *ERC1, WRN, OLIG2, FANCC*, and *ACSL6* in ACC; and *SOX2* and *NDRG1* in ESCA leading in performance with AUCs ranging 0.832-0.911. On the predictability of under-expressed genes, the most notable ones were *RHOA* in THYM (AUC 0.908 ± 0.05), *LSM14A*, *THRAP3*, and *MTOR* in the brain LGG (AUCs ranging from 0.785 to 0.818) and *BAP1* in MESO (AUC 0.818 ± 0.084). The expression status of many genes was predictable across multiple cancer types (Supplementary Fig. 3b).

### Feasibility of predicting protein expression level status with DL

As the next step in our multi-omics pan-cancer study, we assessed the ability of DL to detect histomorphological changes that might be associated with the alterations in the expression of proteins. Towards this end, we trained models to predict the under and/or over-expression status of proteins associated with certain driver genes based on proteomic profiles provided by TCGA. It is worth noting that association with a gene in this context refers to the encoding of a protein by that gene and we use associated with/encoded by interchangeably throughout the paper. A total of 267 and 309 driver genes were qualified to evaluate the predictability of their corresponding under- and over-expression status at the protein level, respectively.

**Fig. 4:**
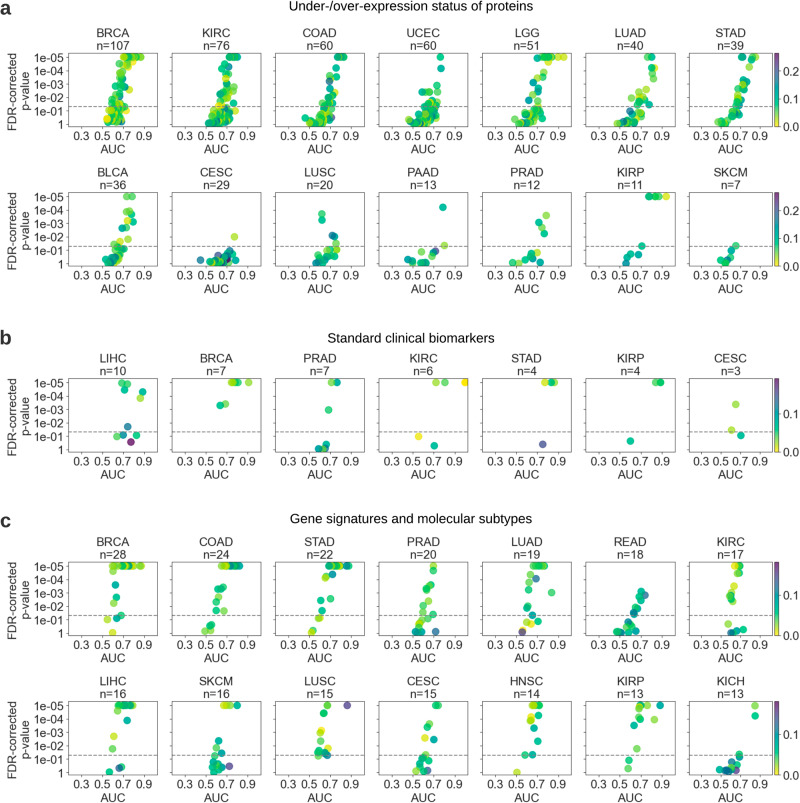
Protein expression status, standard clinical biomarkers, gene signatures, and molecular subtypes could be inferred with deep learning across many cancer types. Scatter plots show the average test area under the curve (AUC) for each model trained to predict (**a**) under-/over-expression status of proteins (**b**) standard clinical biomarkers across selected cancer types, and (**c**) gene signatures and molecular subtypes. Plots are ordered by the number of tested biomarkers in each cancer type. Due to space limitations, cancer types with fewer biomarkers are not shown in this figure but are provided in Supplementary Fig. 9. Please refer to the caption of Fig. 3 for a detailed explanation of the visualization.

We achieved an average AUC of 0.666 (±0.107), with the under-expression status being slightly less predictable on average (mean AUC 0.662, ±0.105) compared to its over-expressed counterpart (mean AUC 0.669 ± 0.109). The expression status prediction of almost all genes under investigation performed above random (Fig. 4a), with more than half of them being detectable with an AUC of at least 0.65 and over 30% of them further achieving an AUC above 0.70. BRCA had the highest predictability rate, where the expression status of 37 out of 107 genes was detectable with an AUC of at least 0.7. It was followed by KIRC and low-grade brain glioma (25 out of 51 and 76 genes, respectively). The expression level status of a large number of proteins encoded by driver genes was highly predictable, with *TFRC*, *ATM*, and *PIK3CA* in low-grade brain glioma; *NRAS*, *FOXO3*, *MYC*, and *TP53* in papillary renal cell carcinoma; *CDKN1B* in HNSC; and *MYC* in SARC, exhibiting the top performance with AUCs ranging 0.835-0.974. Multiple under-expressed proteins were also predictable to a great extent, the top-performing ones being *CASP8*, *MET*, *BCL2*, and *SETD2* in BRCA; *AR* and *TFRC* in gastric cancer; and *VHL* in lung cancer with AUCs ranging 0.814–0.866. We found that the p53 protein over-expression (encoded by *TP53*) was consistently predictable in six of the eight tested cancers, including renal cell carcinomas, lower-grade brain glioma, and endometrial cancer, with AUCs ranging from 0.672 to 0.835. The expression status of many other proteins was also detectable across multiple cancer types (Supplementary Fig. 3c).

### Predictability of transcriptomic and proteomic biomarkers is positively correlated

Despite the average predictability of the protein expression status being higher than that of the transcriptome in the targets we investigated (Table 1), both omic types had around 200 highly predictable genes (i.e., AUC > 0.7). The slightly lower overall performance of transcriptome expression prediction may be attributed to it inherently being less predictable compared to its proteomic counterpart. Considering how predictability changes across the molecular landscape (Supplementary Fig. 4), we measured a positive PCC of 0.227 (*p* < 1e–05) between the transcriptomic and proteomic biomarkers with regard to their over-expression status. A positive linear relationship also existed between genetic alterations and under-expressed transcriptomes (PCC: 0.131, *p* < 0.01) as well as its proteomic counterpart (PCC: 0.068, *p* < 0.01).

### Feasibility of predicting standard clinical biomarkers with DL

We tested the feasibility of DL to predict well-established biomarkers that are routinely used in clinical management. Towards this end, a set of standard of care features was compiled by following the biomarker acquisition approach in ref. ^11^, including data for tumor grade, microsatellite instability (MSI) status (in colorectal and gastric cancer), histological subtypes, hormone receptor status (in BRCA), and Gleason score (in prostate cancer).

Standard pathology biomarkers demonstrated a relatively higher predictability performance, with an average AUC of 0.742 (±0.120). None of the biomarkers had a performance worse than random (i.e., all AUCs > 0.5) and almost 30% of them could be inferred with an AUC of above 0.8, a potential sign of high predictability (Fig. 4b). As expected, histological subtypes were in general highly predictable, especially for BRCA, renal cell carcinomas, hepatocellular and gastric cancer. Prediction of molecular features in clear cell and chromophobe subtypes of renal cell carcinoma had the highest performance, reaching up to an AUC of 0.999. Invasive ductal carcinoma (IDC) and invasive lobular carcinoma (ILC) subtypes of BRCA were well detectable from WSIs, with AUCs ranging 0.759–0.908. Our models were able to predict hormonal receptor status in BRCA, with AUCs of 0.806 and 0.744, for estrogen (ER) and progesterone (PR) receptors, respectively. Notably, multiple clinical biomarkers important for hepatocellular cancer could also be accurately inferred from histology, including growth patterns (AUC up to 0.862) and the etiological status of non-alcoholic fatty liver disease (NAFLD, AUC 0.826 ± 0.054). Another highly predictable biomarker was MSI status, which was detectable in both colon and gastric cancer with average AUCs of 0.716 and 0.773.

### Feasibility of inferring molecular subtypes and gene expression signatures from routine images

To evaluate the capability of DL to detect molecular subtypes and gene expression signatures of cancer from WSIs, we compiled a set of well-established features with clinical and/or biological significance, by closely following the experimental details in a previous study^11^. This includes features that are relevant for prognosis and targeted therapies such as molecular subtypes and clusters, immune-related gene expressions, homologous recombination defects, cell proliferation, interferon-γ signaling and macrophage regulation and hypermethylation/mutation^32–34^. Given their association with higher-level functions, these biomarkers may potentially have a larger impact on the morphology than the previously-assessed alterations, especially compared to single mutations^11^.

Overall, molecular subtypes and gene signatures were considerably predictable with an average AUC of 0.653 (±0.097). Almost half of them were detectable at an AUC level greater than 0.65 (Fig. 4c). Many biomarkers with high AUC were observed in BRCA (18 out of 28 biomarkers) and the adenocarcinomas of the stomach (16 out of 22 biomarkers) and colon (14 out of 24 biomarkers). Our method was capable of inferring TCGA molecular subtypes in multiple cancer types, including KIRP (AUC up to 0.884 ± 0.085), gastric cancer (AUC up to 0.875 ± 0.048), LUSC (AUC up to 0.861 ± 0.015), and BRCA (AUC up to 0.859 ± 0.028). Notably, the average AUC for PAM50 subtypes in BRCA was 0.752 (±0.080), reaching up to 0.871 (±0.015) for the Basal subtype. Consensus molecular subtypes (CMS) in colon cancer (i.e., CMS1, CMS2, CMS3, CMS4) were also potentially detectable with an inter-subtype average AUC of 0.763 (±0.068), reaching up to 0.821 (±0.083) for CMS1. Cell proliferation and hyper-methylation emerged as relatively well-predicted biomarkers, particularly in breast, stomach, colon, and lung cancers, with AUCs reaching up to 0.854.

### Feasibility of inferring clinical outcomes and treatment response from diagnostic histology slides

The ability to accurately estimate prognosis can be vital for clinical management operations. Previous work has focused on developing prognostic models from routine clinical data, standard of care features, histopathological assessment, molecular profiling, and more recently, morphological features acquired via DL^43–47^. There have also been attempts to use ML approaches and image-based features to predict clinical endpoints in different cancers, such as melanoma and non–small cell lung cancer^48,49^. In our study, we explored the end-to-end predictability of prognostic outcomes directly from histology across multiple cancer types by treating the clinical outcome endpoints such as OS, DSS, DFI, and PFI as potential prognostic biomarkers^35^. We further expanded our analysis towards detecting treatment responses directly from WSIs to assess whether DL models can correlate histomorphological features with the outcome of a therapy or drug. To the best of our knowledge, this study constitutes the first systematic attempt to assess the DL-based predictability of drug responses across multiple cancer types.

**Fig. 5:**
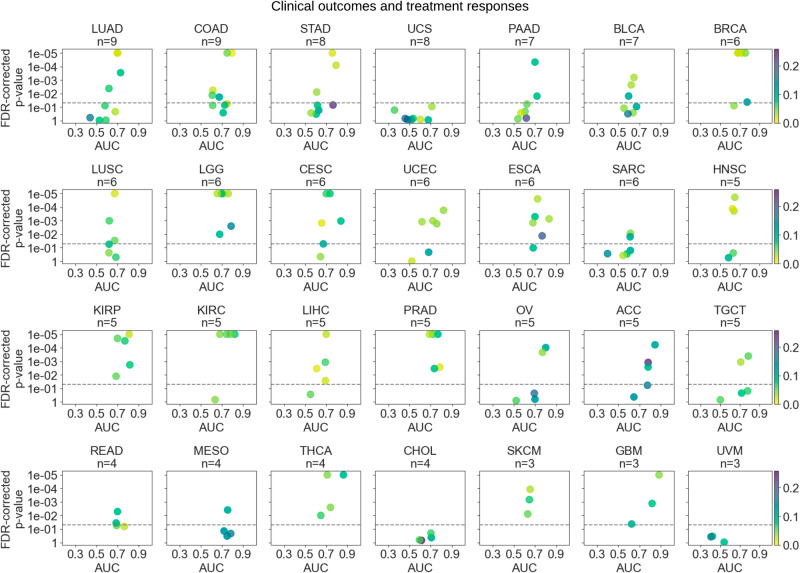
Deep learning could infer clinical outcomes and treatment responses from diagnostic histology slides. Scatter plots show the average test area under the curve (AUC) for each model trained to predict clinical outcomes and treatment responses across selected cancer types. Plots are ordered by the number of tested biomarkers in each cancer type. Due to space limitations, cancer types with fewer biomarkers are not shown in this figure, but are provided in Supplementary Fig. 9. Please refer to the caption of Fig. 3 for a detailed explanation of the visualization.

Overall, the predictive performance of clinical outcomes and treatment responses was considerably high, with a mean AUC of 0.671 (±0.12). Almost 40% of the tested targets were predictable at an AUC level of 0.70 or above (Fig. 5). We acquired the best overall performance in GBM, ACC, and KICH with mean AUCs of 0.77. They were followed by renal papillary cell carcinoma, MESO, THCA, prostate cancer, renal clear cell carcinoma, and ESCA, with overall AUCs ranging from 0.731 to 0.76. OS in KICH, GBM, THCA, and ACC; DFI in ESCA and renal clear cell carcinoma; DSS in GBM; residual tumor status in endometrial carcinoma, and the treatment response in papillary renal cell carcinoma were among the top-performing targets with AUCs ranging from 0.815 to 0.924.

Among the 20 drugs we investigated in our study, DL was able to predict the response in half of them with an AUC of at least 0.7. Cisplatin was the most notable drug with AUCs ranging 0.763–0.837 in cervical, testis and gastric cancers. The other predictable drugs were temozolomide in LGG; paclitaxel in BRCA; leucovorin, oxaliplatin, and fluorouracil in colon cancer; etoposide in testis cancer; and gemcitabine in pancreatic cancer.

### Pan-cancer predictability is consistent across different datasets

To show that a comparable performance can be achieved across different datasets, we repeated our experiments using publicly available data from the CPTAC. We limited our analysis to the prediction of the presence of SNVs in driver genes due to both TCGA and CPTAC relying on the same set of driver genes, hence exhibiting a relatively large overlap. A total of 176 driver genes across seven cancer types were identified that have available mutation data in both datasets. The investigated cancers were endometrial carcinoma (*n* = 61), PDA (*n* = 4), LUSC (*n* = 21), LUAD (*n* = 14), head and neck cancer (*n* = 12), GBM (*n* = 4), and colon adenocarcinoma (*n* = 60). Comparable overall performance was observed on both datasets across almost all tested cancer types (Supplementary Fig. 5, *p* > 0.05 under two-sided t-test for all but COAD) with within-cancer average AUCs ranging 0.578–0.655 and 0.567–0.672 for TCGA and CPTAC, respectively. Performance across biomarkers varied more for CPTAC cohorts, as indicated by the shape of violin plots. Overall, our results indicate that the predictability of the biomarkers is consistent and dataset-independent.

## Discussion

This study assessed the general feasibility of predicting a plethora of biomarkers from a pan-cancer perspective, using DL and histomorphological features extracted from H&E-stained diagnostic slides. Morphological characteristics captured from histology with an encoding-decoding DL model were used for inferring biomarkers across the omics spectrum. We observed a notable performance for certain genetic alterations (e.g., *TP53*) and clinically-relevant markers (e.g., standard-of-care features and molecular subtypes) across multiple cancer types. The biomarker-level performance seemed to exhibit a reasonable degree of consistency, as the cross-validated models of a biomarker performed mostly similarly. A comparable predictive performance was obtained to a certain degree when experiments were repeated on an independent dataset, further showing the overall capability of DL for molecular profiling across multiple cancer types.

The performance of a biomarker did not seem to depend on the factors intrinsic to the tested populations, such as the number of cases (i.e., population size) and the proportion of positive-negative samples in the whole population (Supplementary Fig. 6a, Supplementary Fig. 7). We argue that the dataset size is more important for the stability of the results, rather than the performance itself, i.e., having a larger training subset is likely to yield models with more consistent performance. In addition, our biomarkers typically had an unbalanced distribution, due to most of the biomarkers having a much smaller number of positive cases than that of negative. We tackled this problem at training time by oversampling more from the underrepresented class and later assessed its impact on the stability of performance. This was achieved by examining the sample size and class ratios in comparison to the range of AUCs across the cross-validation folds for each biomarker (measured by standard deviation). Our observation revealed a negative relationship for both factors (Supplementary Fig. 6b). This may indicate that the performance is likely to become less variable with an increasing number of samples and a more balanced dataset. In addition, we performed an experiment to assess the relationship between the biomarker predictability and tumor purity (Supplementary Fig. 1, Methods: Tumor purity experiment). Our analysis revealed that predicting biomarker status solely based on tumor purity as an independent variable was not feasible. However, we observed some correlation between tumor purity and the predictive performance for certain biomarkers. This correlation may be attributed to the tendency for larger tumor compositions to enhance performance to some extent.

In our study, we found that detecting alterations from histology at different levels of the omics landscape was mostly feasible for the majority of the investigated genes. Certain mutations likely to be associated with poor clinical outcomes showed a decent performance across multiple cancer types, notable examples being those harbored by *TP53, BAP1, MTOR*, and *GNAS*. The relatively high and consistent performance observed for *TP53* might be attributed to tumors with TP53 mutations likely being poorly differentiated, and exhibiting visually discernible higher-grade cell changes^15^. Identifying patients with certain alterations is critical for precision treatment and can pave the way to developing targeted therapies. For instance, recent studies show that the detection of mutations in *BAP1* can potentially be useful for the development of targeted treatment strategies in KIRC^50^. Similarly, *MTOR* mutations can serve as biomarkers for predicting tumor responses to mTOR inhibitors, which are already being used to treat human cancer^51^. One of the highly predicted genes, *GNAS*, is known to promote cell proliferation and migration in BRCA when expressed at high levels, and thus, can potentially be used as a therapeutic target^52^.

The identification of downstream changes in tumors and their differentiation from normal cells can aid in uncovering the complex mechanisms that influence the anticancer drug response and potentially enhance the prediction of therapeutic outcomes^53–56^. Despite many studies targeting tumor metabolism, the attempts to assess the detectability of transcriptomic and proteomic changes from WSIs have been highly limited^15,57,58^. In our study, we observed the possibility of predicting the under-/over-expression status of transcriptomes and proteins to some extent. For instance, we found that p53 over-expression was consistently predictable across multiple cancer types.

Our findings indicated that DL can potentially infer well-established standard-of-care clinical biomarkers, gene expression signatures, and molecular subtypes from histopathological images. Some of these highly predictable targets have already been adopted as actionable biomarkers in clinical practice. This correlation is reasonable since alterations that fundamentally impact tumor biology also often coincide with changes in morphology, making these biomarkers valuable targets for effective treatment strategies. Our findings largely align with those obtained in the recent pan-cancer study conducted by ref. ^12^. The similarities between the two studies enable us to corroborate their findings, and our results offer additional evidence for the feasibility of detecting molecular biomarkers from histology.

Classification of residual tumors is a critical stage for the course of treatment and is considered an important prognostic biomarker^36^. In our analysis, we found that DL could potentially detect the occurrence (or lack thereof) of residual tumors in multiple cancers, which might indicate that some visual clues correlated with complete remission after treatment are likely to be present in histomorphology at the time of diagnosis. While other clinical outcomes, such as OS and disease-specific survival (DSS), were also predictable to a certain extent, one should note that the definition of clinical outcomes may not always be accurate, especially for the cancer types that need longer follow-up times, have a small cohort size or have a limited number of events^35^. This study assessed the predictability of drug responses from H&E-stained images and revealed that the complete response to several drugs such as cisplatin, temozolomide, and paclitaxel could potentially be detected with DL. These findings suggest that DL approaches hold promise for precision medicine, enabling oncologists to select treatments that may work best for patients by analyzing routine histology slides.

One of the limitations of our study is that we placed specific limitations on biomarker acquisition to maintain a manageable scope for the research. For instance, we restricted the computation of multi-omic biomarker profiles to driver genes and limited our analysis to single variant mutations when considering genomic alterations. Consequently, comparing the predictability of biologically different targets is not possible, as the analysis focused on only a subset of alterations rather than examining all possible variations. Another limitation of the study comes directly from the data itself. While we compared predictability across multiple cancers and omic types, all biomarkers were tested under different sample sizes and prevalence conditions. Many potential biomarkers were simply discarded during data acquisition, as they did not contain enough positive samples. It is important to acknowledge that TCGA is known to have site-specific fingerprints inherent in digital slides, which could bias the accuracy of predictive models. To mitigate this concern, more sophisticated splitting techniques may be considered^59^. We assessed the model performance with AUC, the most commonly used evaluation metric in the presence of class imbalance. However, in scenarios where only a few examples are available for the minority class (such as rare mutations), AUC values can be less reliable. Performance as estimated by AUC can drastically change based on correct and incorrect predictions of the minority class. One should note this inherent drawback when interpreting the results of biomarkers with very small sample sizes and low prevalence. AUC values, class prevalence, validation sample size, corrected *p*-values, and other relevant information for all models are given in Supplementary Data 1.

**Fig. 6:**
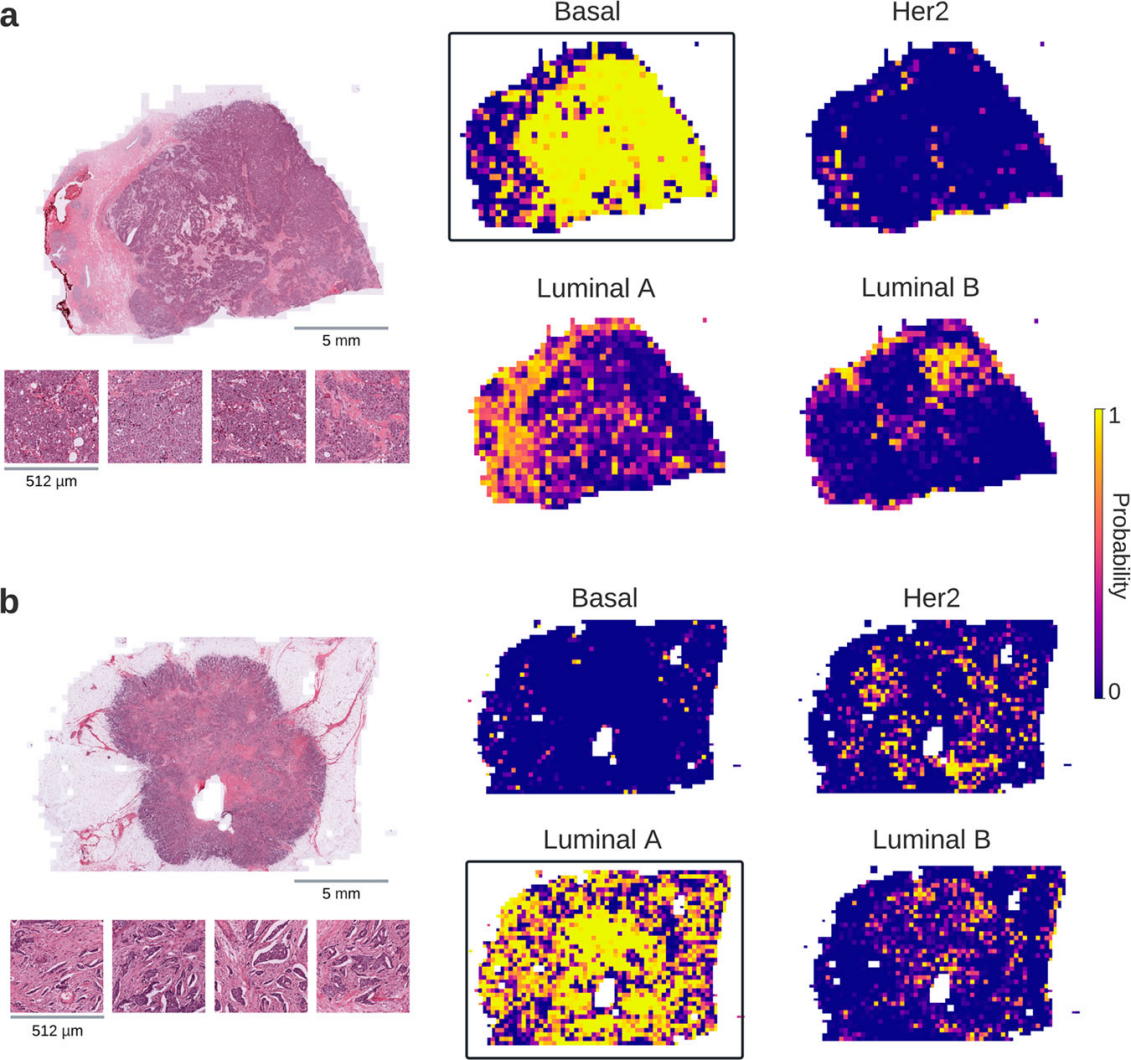
Visualization of predictability with deep learning from histopathological images. Deep learning (DL)-based predictions for the molecular subtypes of breast cancer (i.e., Basal, HER2, Luminal A, and Luminal B) are visualized for two selected patients using heatmaps. The correctly-predicted subtype in each case is enclosed with a rectangle and the highest-ranking tiles from that class are given alongside the original whole slide image (WSI). The Basal type (**a**) shows sheets of tumor cells without any discernible gland formation, while the Luminal A patient’s tumor (**b**) is composed of well-formed glands. Considering the heatmaps in both cases, one can notice that DL models can identify spatial regions that are relevant to the target class. Scale bar for WSIs: 5 mm. Scale bar for tiles: 512 µm.

Exploring the black-box representation of DL models can be useful in revealing the morphological patterns that may be linked to certain alterations or phenotypic outcomes^11,14^. One way to visualize the spatial regions that are critical for inferring a biomarker status is superimposing the tile-level prediction scores (i.e., probabilities) onto WSIs to create spatial heatmaps (Fig. 6). The highest-ranking tiles within these heatmaps represent the visual characteristics learned by the DL model to solve the prediction task at hand. For instance, different types of breast tumors may show distinct differences in morphology, which can be identified by DL and utilized to differentiate specific subtypes (Fig. 6a, b). Similarly, top-ranked tiles from CMS of colon cancer (Supplementary Fig. 8a) show distinct morphological patterns that consistently align with the histopathology of CMS subclasses as shown in previous studies^11,19^. In addition, morphological traits associated with MSI unstable cases, e.g., containing large amounts of tumor-infiltrating lymphocytes, can be seen in the highest-ranking MSI tiles acquired from patients with colon and gastric cancers (Supplementary Fig. 8b–c). While DL can identify clinically-relevant morphological features, it may also be useful to trace back the visual patterns that are associated with molecular alterations. For instance, highly-predicted tiles from a mutated *BRAF* case in papillary THCA show distinct histological features compared to its wild-type counterpart (Supplementary Fig. 8d). In addition, DL may be a useful tool to explore visual characteristics with unknown links to histomorphology. For instance, no distinct features are known to distinguish the mutated *TP53* and its wild-type in BRCA, but DL can still pick tumor tiles that show different visual characteristics for both classes (Supplementary Fig. 8e). This may provide insights to better understand the potential impact of this alteration on cancer cell morphology.

Showing the overall feasibility of predicting multi-omic biomarkers with DL marks an important step in pursuit of achieving end-to-end detection systems from histology, which can potentially assist clinicians in patient management, accelerate diagnosis, and help develop more patient-centric treatments. The approach also presents an opportunity to apply multi-omic biomarkers in a rapid and low-resource setting without requiring time-consuming and expensive biological tests. While this study has elucidated early observations on the factors determining a biomarker’s predictability, further understanding would be necessary before the mainstream adoption of DL-based methods for multi-omic biomarker profiling from standard tissue imaging in day-to-day clinical settings^60^.

A systematic analysis of the model’s generalizability is crucial to comprehend the true scope of predictability. Additionally, there is still a need for further investigation into the specific mechanisms of biomarker detectability. The results presented in this study provide an opportunity to identify promising biomarkers for clinical adoption and those that require re-evaluation. For example, biomarkers with low AUC can be re-trained with a larger dataset and customized modeling to confirm whether their poor performance is due to limited biological signals or a model’s inability to capture them due to suboptimal configuration. Conversely, the biomarkers with high AUC can undergo rigorous validation to further assess their immediacy to clinical adoption. This may include assessment on multiple external datasets, comparison with standard care approaches, concordance with treatment response, prospective evaluation, and a cost-effectiveness analysis for clinical implementation, considering potential benefits and resource implications^20^.

Future work will explore the internal representations of the predictive models to reveal potential associations between omics, tumor morphology, and model predictions. This exploration may provide insights into understanding the varying performance of predictability across different omics. While this study formulates the problem of predicting biomarker status from H&E-stained images as a single-task classification problem, future studies should investigate explicit multi-task methods. Considering that certain biomarkers may be correlated with each other, employing such methods may potentially improve predictability performance.

### Supplementary information

Supplementary Information

Description of Additional Supplementary Files

Supplementary Data 1

Reporting Summary

## Data Availability

TCGA whole slide images are available at https://portal.gdc.cancer.gov/. Genetic, transcriptomic, proteomic, and clinical data used to generate biomarker profiles for cases in the TCGA cohorts are available at https://portal.gdc.cancer.gov/ and https://cbioportal.org/. Clinically relevant driver genes are available at https://cancervariants.org/. CPTAC whole slide images are available at https://wiki.cancerimagingarchive.net/display/Public/CPTAC+Imaging+Proteomics/. Genetic data used to generate biomarker profiles for cases in the CPTAC cohorts are available at https://portal.gdc.cancer.gov/. The source data used to generate the figures, including AUC values, class prevalence, validation sample size, corrected *p*-values, and the other relevant information for the models evaluated in the study, are given in Supplementary Data 1.

## Code availability

Codes used for the analysis are available in the following URL: https://github.com/Panakeia-Technologies/Multiomics-PANCancer (10.5281/zenodo.6566146)^61^. The experiments and analyses in this work were carried out with python 3.6 and the following python libraries: torch (1.9.0), torchvision (0.10.0), google-cloud-storage (1.32.0), openslide-python (1.1.1), pillow (6.0.0), opencv (3.4.2), tensorboard (1.15.0), numpy (1.18.1), pandas (1.3.5), seaborn (0.11.0), scipy (1.4.1), scikit-learn (0.22.1), statsmodels (0.11.0), and matplotlib (3.5.1).
