# Supplementary material for: A systematic pan-cancer study on deep learning-based prediction of multi-omic biomarkers from routine pathology images
Source: Commun Med (Lond). 2024 Mar 15;4:48. doi: 10.1038/s43856-024-00471-5 (PMC10942985; doi:10.1038/s43856-024-00471-5)
Supplement: Supplementary file 1 — Supplementary Information [file 43856_2024_471_MOESM1_ESM.pdf]

A systematic pan-cancer study on deep learning-based prediction of multi-omic biomarkers from routine pathology images

Salim Arslan★, Julian Schmidt, Cher Bass, Debapriya Mehrotra, Andre Geraldes, Shikha Singhal, Julius Hense, Xiusi Li, Pandu Raharja-Liu, Oscar Maiques, Jakob Nikolas Kather, Pahini Pandya

Supplementary Information

Table of Contents 1

Supplementary Figures 2

- Supplementary Fig. 1: Analysis of tumor purity as a biomarker predictor and its relationship to prediction performance 2
- Supplementary Fig. 2: Average performance for each cancer across all biomarker types 4
- Supplementary Fig. 3: Biomarkers that were predictable across multiple cancers 5
- Supplementary Fig. 4: Cross-correlation of the detectability of molecular alterations across genomic, transcriptomic, and proteomic biomarkers 6
- Supplementary Fig. 5: Reproducibility of pan-cancer predictability on external dataset 7
- Supplementary Fig. 6: Impact of sample size and positive class ratio on the prediction performance 8
- Supplementary Fig. 7: Average population size and class ratio across all cancer and biomarker types. 9
- Supplementary Fig. 8: Highest scoring tiles from selected biomarkers in colon, gastric, thyroid, and breast cancers. 10
- Supplementary Fig. 9: Performance of individual biomarkers across selected cancer types 11

Supplementary Tables 12

- Supplementary Table 1: Number of images and patients included in the study. 12
- Supplementary Table 2: Number of images and patients included in the CPTAC dataset. 13
- Supplementary Table 3: Details of the binarization rules used to define actionable survival outcomes. 14
- Supplementary Table 4: Average performance and standard deviation for all cancer types. 15

Supplementary References 16

★: Correspondence to: salim@panakeia.ai

Supplementary Figures

Supplementary Fig. 1: Analysis of tumor purity as a biomarker predictor and its relationship to prediction performance

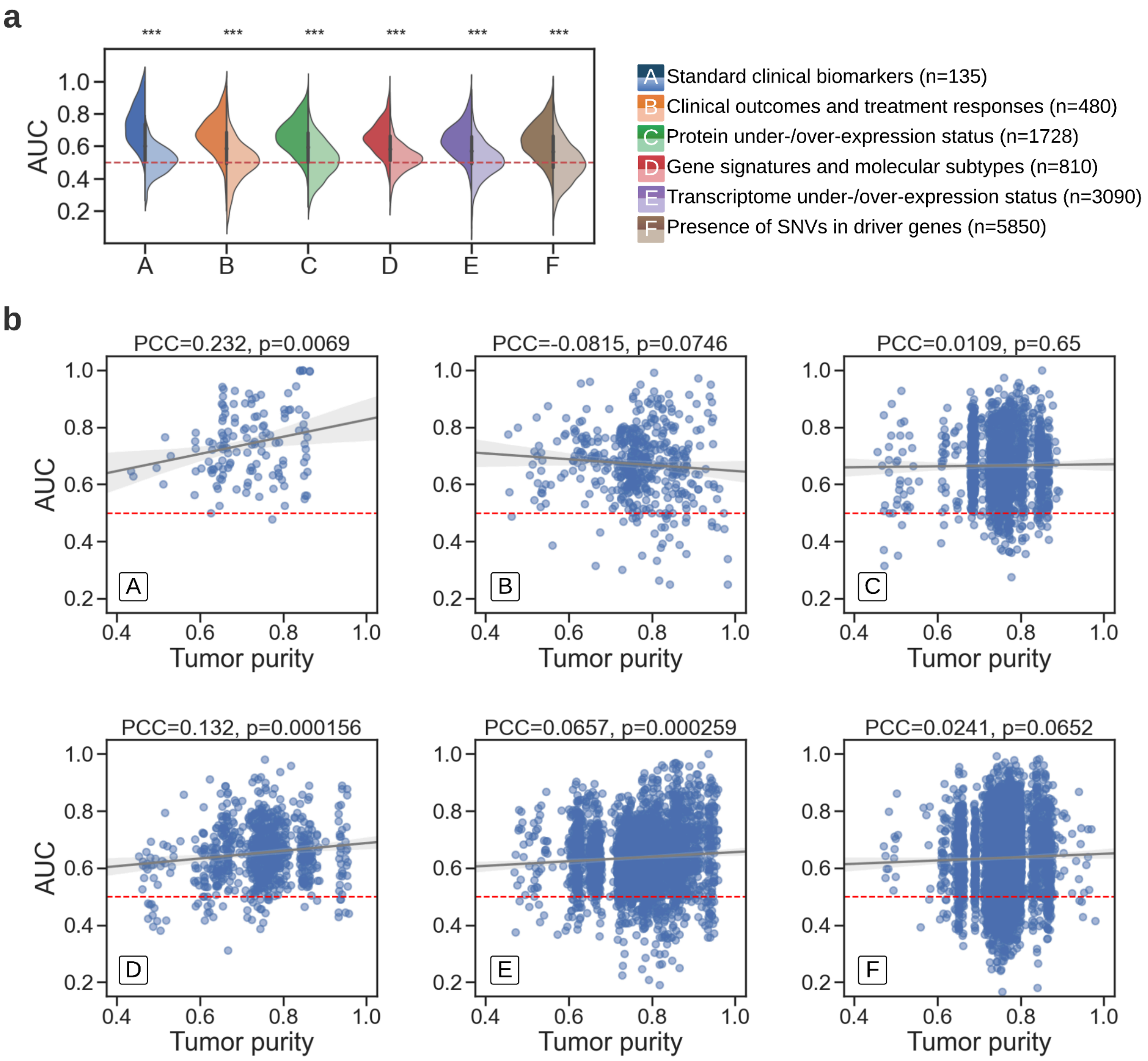

We assessed the relationship between biomarker predictability and tumor purity by **(a)** devising a classification task to directly predict biomarker status using tumor purity and **(b)** performing a correlation analysis between tumor purity and predictability from hematoxylin and eosin (H&E)-stained slides with deep learning (DL), which is measured by the area under the curve (AUC). For the classification task in (a), we adopted a similar experimental setup. Still, instead of image-based features, we used tumor purity as a predictor and a random forest as a binary classifier (Methods: Tumor purity experiment). Violin plots in (a) show the AUC distribution across all tested biomarker categories (with SNVs referring to single nucleotide variants), where the left half of each violin represents the AUC values obtained from the DL models as previously given in Fig. 2b, and the right half corresponds to the AUC distribution of tumor purity-driven random forest classifiers, with asterisks indicating the statistical significance of the difference between the two, where $p < 1e\text{-}05$ for all subgroups. Overall, performance was no better than random across any of the biomarker

categories when tumor purity was used as a predictor. This may indicate that tumor purity alone is not a strong variable for predicting biomarker status. After observing a random performance in (a), we further measured the degree of a potential relationship between tumor purity and the performance of the DL-based predictive models. The percentage of tumor cells used in (a) was averaged across samples for each biomarker. Scatter plots in (b) show how the average tumor purity is correlated with the AUC of the DL models for each biomarker category as measured by the Pearson correlation coefficient (PCC). The dotted line marks the AUC at 0.5 and the solid lines correspond to regression estimates for tumor purity and AUC, with the shaded area showing the size of their confidence interval. Overall, we found a statistically significant positive correlation ($p < 0.05$) for (A) standard clinical biomarkers (PCC=0.232), (D) gene signatures and molecular subtypes (PCC=0.132), and (E) the under-/over-expression of transcriptomes (PCC=0.066). For other biomarker groups, there was no statistical significance ($p > 0.05$). This might indicate that tumor purity can positively impact the predictability to some extent for certain biomarker types.

Supplementary Fig. 2: Average performance for each cancer across all biomarker types

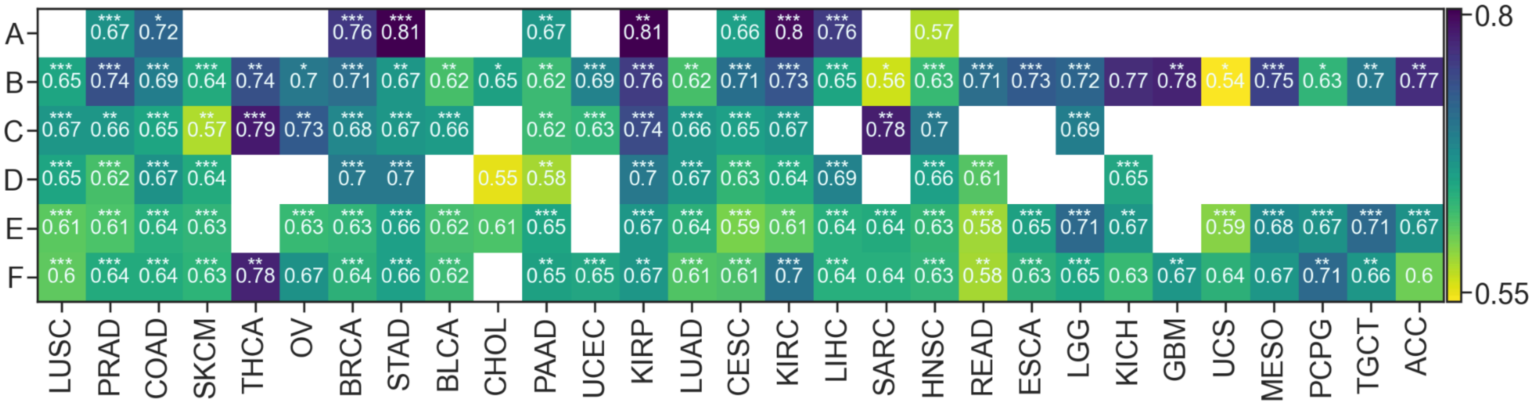

Average area under the curve (AUC) across all cancers confounded by biomarker type is shown in a heatmap. Empty cells correspond to having no data for those cancer-biomarker groups. Asterisks within heatmap cells indicate the statistical significance of the performance difference between AUC values of a subgroup against randomly sampled values of the same underlying distribution (no asterisk: not significant (n.s.), *: $p < 0.05$, **: $p < 0.01$, ***: $p <$ 1e-05). Cancer abbreviations are defined in Supplementary Table 1. Lymphoid neoplasm diffuse large B-cell Lymphoma (DLBC), uveal melanoma (UVM), and thymoma (THYM) were excluded from the analysis due to constituting only a few targets. The following coding was used to abbreviate the biomarker types: **A** for standard clinical biomarkers; **B** for clinical outcomes and treatment responses; **C** for under-/over-expression of proteins; **D** for gene signatures and molecular subtypes; **E** for under-/over-expression of driver genes; and **F** for the presence of single nucleotide variants (SNVs) in driver genes. Overall, we obtained a better-than-random average performance across all biomarker types (i.e. mean AUC > 0.5). Among the cancer types targeting standard clinical features **(A)**, kidney renal papillary cell carcinoma (KIRP, AUC: 0.805 ±0.132, $p < 0.01$) and stomach cancer (STAD, AUC: 0.805 ±0.084, $p <$ 1e-05) had the top average performance, followed by clear cell renal cell carcinoma (KIRC), breast adenocarcinoma (BRCA), and colon cancer (COAD), each with a mean AUC over 0.7. Multiple cancer types showed a relatively good performance with average AUCs above 0.7 especially when considering the predictability of the clinical outcomes and treatment responses **(B)**, where the performances of such predictions were among the highest across studies. Among them, the most notable studies were kidney renal papillary cell carcinoma (KIRP), adrenocortical carcinoma (ACC), glioblastoma multiforme (GBM), and kidney chromophobe (KICH) with average AUCs reaching as high as 0.777. For genomic, transcriptomic, and proteomic biomarkers **(C, E, F)**, the performances within individual cancer types were primarily consistent with their corresponding general trend. The highest performances were observed in thyroid carcinoma (THCA) and sarcoma (SARC) for the prediction of proteomic expression status with average AUCs around 0.78; in lower-grade glioma (LGG) and testicular germ cell tumors (TGCT) for the predictability of transcriptomic biomarkers with AUCs slightly above 0.7; and in kidney renal clear cell carcinoma (KIRC), pheochromocytoma/paraganglioma (PCPG), and thyroid carcinoma (THCA) for the detection of genetic alterations in driver genes with average AUCs ranging from 0.705 to 0.779. Top-performing cancers from the gene signatures and molecular subtypes **(D)** were breast cancer (BRCA), gastric cancer (STAD), and kidney renal papillary cell carcinoma (KIRP), each having an average AUC of 0.7.

Supplementary Fig. 3: Biomarkers that were predictable across multiple cancers

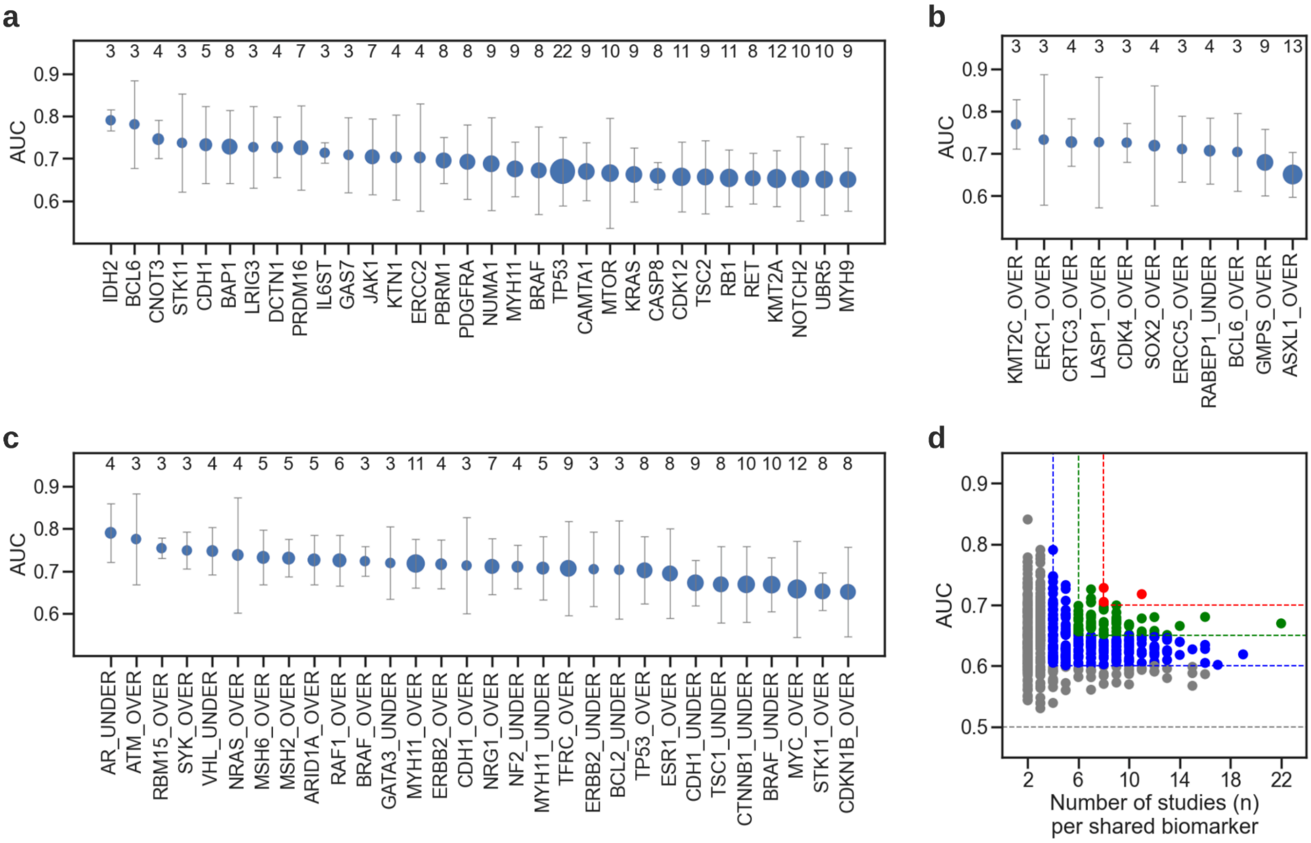

Presence of single nucleotide variants (SNVs) and under-/over-expression of transcriptomes and proteins can be predicted in at least three and seven cancer types with an average area under the curve (AUC) of 0.7 or 0.65, respectively. The size of a marker represents the frequency of a biomarker. Error bars show the standard deviation of AUC across cancers. The number of appearances (n) of a biomarker across cancers is shown in the secondary x-axis. **(a)** Alterations in *TP53* were detectable in almost all cancers, with 7 of them having an AUC of at least 0.7 and 14 of them showing AUCs greater than 0.65. Other genes with a cross-cancer AUC of at least 0.7 were *BAP1* (predictable in 8 cancers), *PRDM16* and *JAK1* (predictable in seven cancers), and *CDH1* (predictable in five cancers). *CDK12, RB1, MTOR, NOTCH2, UBR5, and KMT2A*, are also worth mentioning with their mutations being detectable in at least 10 different cancers with a mean AUC of 0.65. **(b)** Over-expression of *KMT2C* had a consistently high prediction rate with AUCs ranging from 0.733-0.837 in kidney renal papillary cell carcinoma, ovarian serous cystadenocarcinoma, and testis cancer. Other notable genes that were detectable across multiple cancer types at over-expression levels were *ERC1, CRTC3, LASP1, CDK4, SOX2, ERCC5, BCL6,* and the under-expression status of *RABEP1*, each being predicted in three or more different cancers. **(c)** Over-expression of *MYH11* was detected in 7 out of 11 cancer types with AUCs ranging from 0.705 to 0.809. Other notable genes associated with protein over-expression were *TFRC, TP53, MSH2, MSH6, ARID1A, RAF1*, and NRG1, showing a high predictability in at least five malignancies, with AUCs reaching 0.942. Under-expression of proteins encoded by *MYH1, NF2, VHL,* and *AR* were also predictable in at least four cancers, with AUCs reaching 0.856. **(d)** Scatter plot showing the AUCs of genetic alterations, as well as transcriptome and protein expression status predictable in multiple cancer types. Areas outlined with red, blue, and green lines mark the zones with high predictability and frequency of appearance. Red points are the biomarkers with an AUC of at least 0.7 and a frequency of eight and above. Green points correspond to the biomarkers with an AUC of at least 0.65 and a frequency of six and above. For blue, AUC and frequency are limited to 0.6 and four, respectively.

Supplementary Fig. 4: Cross-correlation of the detectability of molecular alterations across genomic, transcriptomic, and proteomic biomarkers

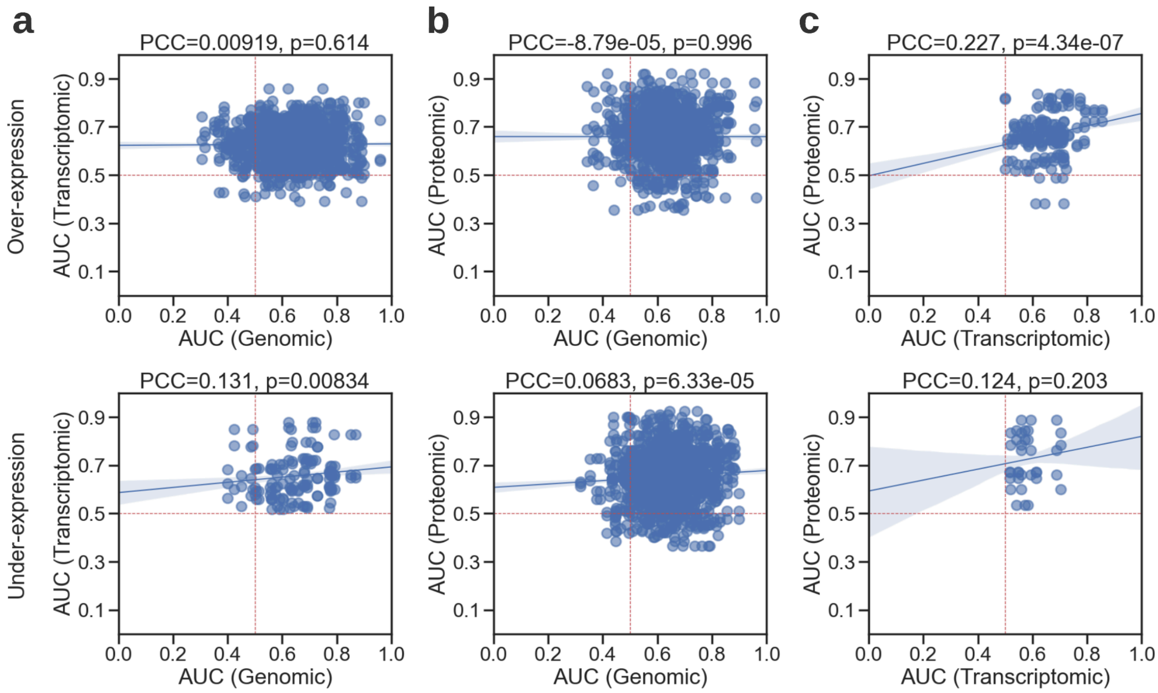

For this analysis, driver genes with a valid genomic, proteomic, and transcriptomic profile across all cancer types were identified and their area under the curve (AUC) values are cross-correlated with the Pearson correlation coefficient (PCC). Since there existed three models per biomarker, each model in a given omic type was compared to all the three models in the other omic type, yielding nine comparisons per gene. Scatter plots show the relationship between AUCs of different omics. The dotted red lines in each plot mark the AUC at 0.5. The solid lines correspond to regression estimates, with the shaded area showing the size of their confidence interval. (**a-b**) Both at the transcriptomic and proteomic expression levels there was no correlation between the predictability of genetic alterations and over-expression status associated with them. Under-expressed transcriptomes and proteins showed a low, but statistically significant positive correlation with genetic alterations, with a PCC of 0.131 ($p < 0.01$) and 0.069 ($p < 0.01$), respectively. **(c)** We measured a positive correlation of 0.227 ($p <$ 1e-05) between the transcriptomic and proteomic biomarkers with regard to their over-expression status.

Supplementary Fig. 5: Reproducibility of pan-cancer predictability on external dataset

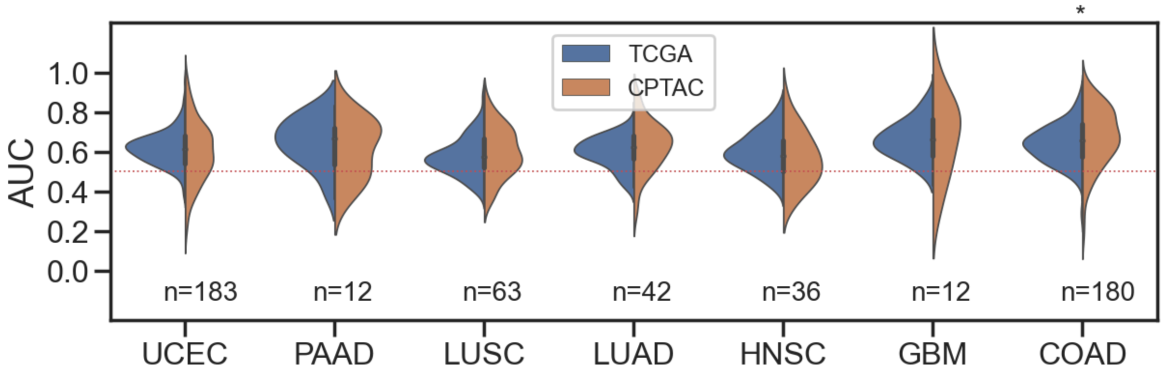

We repeated our experiments using the Clinical Proteomic Tumor Analysis Consortium (CPTAC) data, to show that a comparable performance can be achieved on a different dataset. This data is only available for evaluating the predictability of single nucleotide variants (SNVs) due to both the Cancer Genome Atlas (TCGA) and CPTAC cohorts relying on the same set of driver genes, hence exhibiting a relatively large overlap. A total of 176 driver genes (corresponding to 528 models) across seven cancer types had qualified mutation data in both datasets. The number of models validated per cancer type is shown under each violin plot for each cohort. The investigated cancers were uterine corpus endometrial carcinoma (UCEC), pancreatic ductal adenocarcinoma (PAAD), lung squamous cell carcinoma (LUSC), lung adenocarcinoma (LUAD), head and neck cancer (HNSC), glioblastoma multiforme (GBM), and colon adenocarcinoma (COAD). An asterisk atop a violin plot indicates the difference between the area under the curve (AUC) values of two cohorts being statistically significant (i.e. $p < 0.05$). Here, all biomarkers but those from COAD had comparable AUC distributions in TCGA and CPTAC cohorts. It is important to note that, this experiment was not designed to assess the reproducibility of models trained on one set (e.g. TCGA) and independently tested on another (e.g. CPTAC). It is to show the feasibility of predictability for certain biomarkers regardless of the source of the underlying data.

Supplementary Fig. 6: Impact of sample size and positive class ratio on the prediction performance

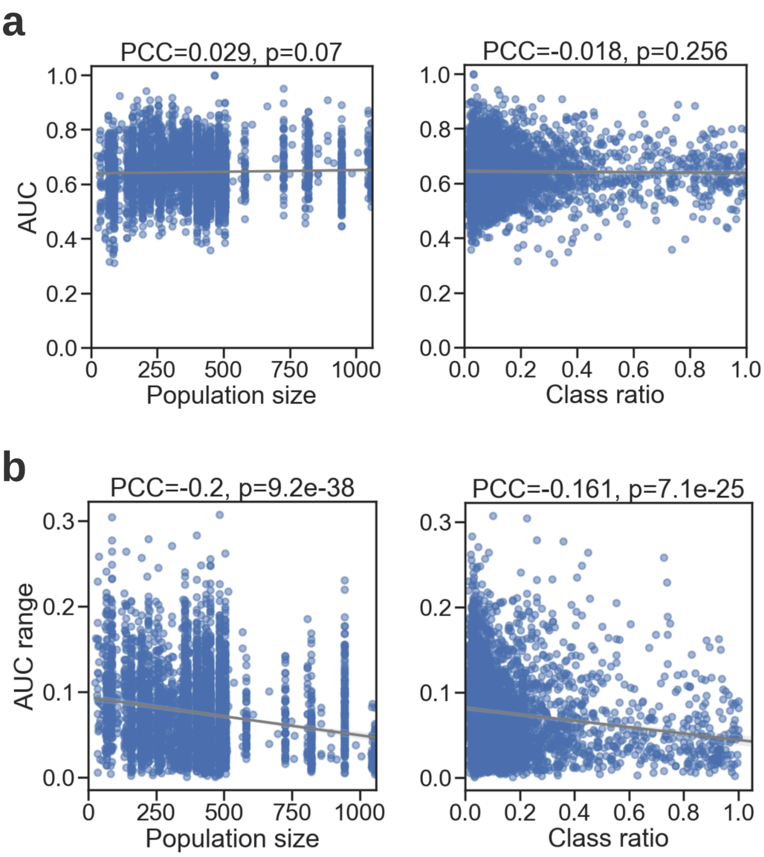

We evaluated the potential influence of the number of samples and the class proportions on the prediction performance, by correlating population sizes and class ratios with **(a)** the biomarker area under the curve (AUC) values and **(b)** the variability in biomarker performance as measured by the standard deviation across the AUCs of cross-validation folds. Population size corresponds to the number of total samples per biomarker and the class ratio is computed as the size of the underrepresented class over the size of other class (i.e. a ratio close to 1 denotes a perfectly balanced class distribution, whereas a ratio close to 0 means a severely unbalanced dataset). We used Pearson's correlation coefficient (PCC) to assess the linear relationship between these numerical variables. Considering the number of steps involved in biomarker acquisition for each omic type and the diverse number of diagnostic slides available for each cancer (Supplementary Table 1), the population size and class distributions per biomarker varied quite significantly across different malignancies (Supplementary Fig. 7). The solid lines in the plots correspond to regression estimates of the x and y variables, with the shaded area showing the size of their confidence interval. **(a)** The Pearson correlation coefficient (PCC) between the population size and the AUC values was 0.029 ($p > 0.05$), indicating no relationship between the two variables. Similarly, a statistically insignificant PCC of -0.018 ($p > 0.05$) was obtained between the class ratio and performance. **(b)** A negative relationship was observed for the AUC variability when it was correlated with population size (PCC: -0.200, $p <$ 1e-05) and class ratios (PCC: -0.161, $p <$ 1e-05). The consistent decrease in variability suggests a more stable and robust performance trend, potentially indicating improved performance with an increasing number of samples and a more balanced dataset.

Supplementary Fig. 7: Average population size and class ratio across all cancer and biomarker types.

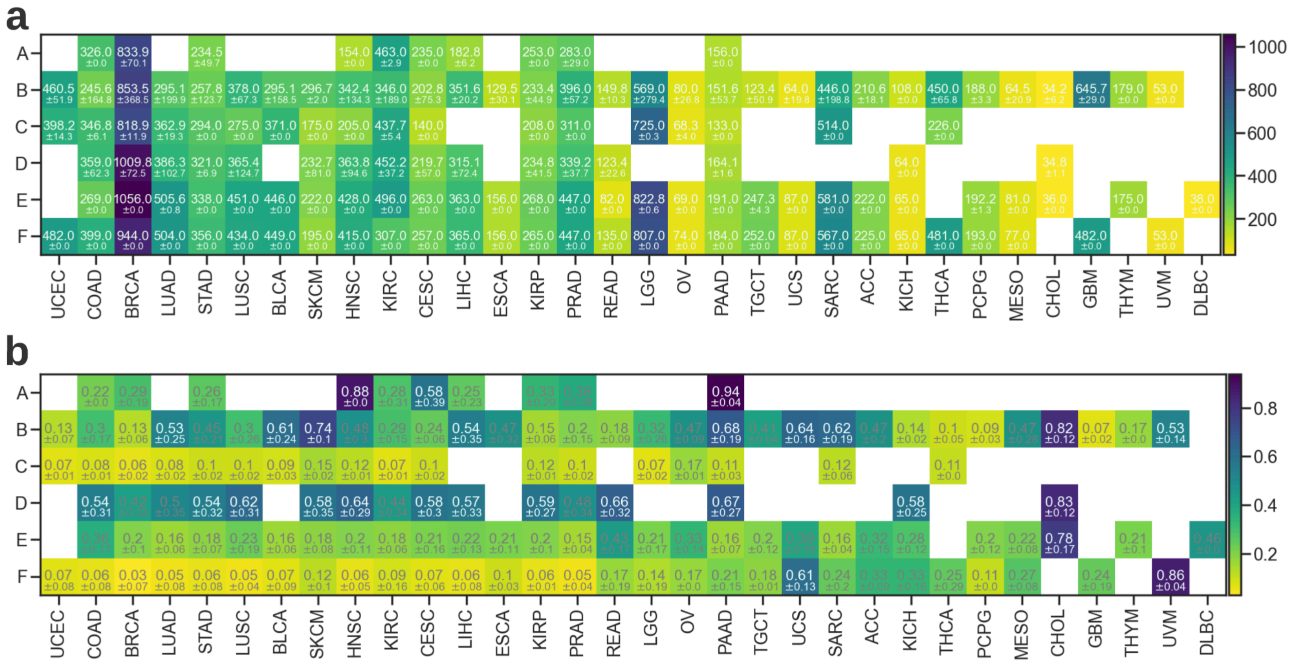

(a) Average population size and **(b)** average class ratio across all cancer and biomarker types are shown in a heatmap. Population size corresponds to the number of total samples per biomarker and the class ratio is computed as the size of the underrepresented class over the size of the other class (i.e. a ratio close to 1 denotes a perfectly balanced class distribution, whereas a ratio close to 0 means a severely unbalanced dataset). Empty cells indicate no data for those cancer-biomarker groups. The following coding was used to abbreviate the biomarker types: **A** for standard clinical biomarkers; **B** for clinical outcomes and treatment responses; **C** for under-/over-expression of proteins; **D** for gene signatures and molecular subtypes; **E** for under-/over-expression of driver genes; and **F** for the presence of single nucleotide variants in driver genes. Cancer abbreviations are defined in Supplementary Table 1.

Supplementary Fig. 8: Highest scoring tiles from selected biomarkers in colon, gastric, thyroid, and breast cancers.

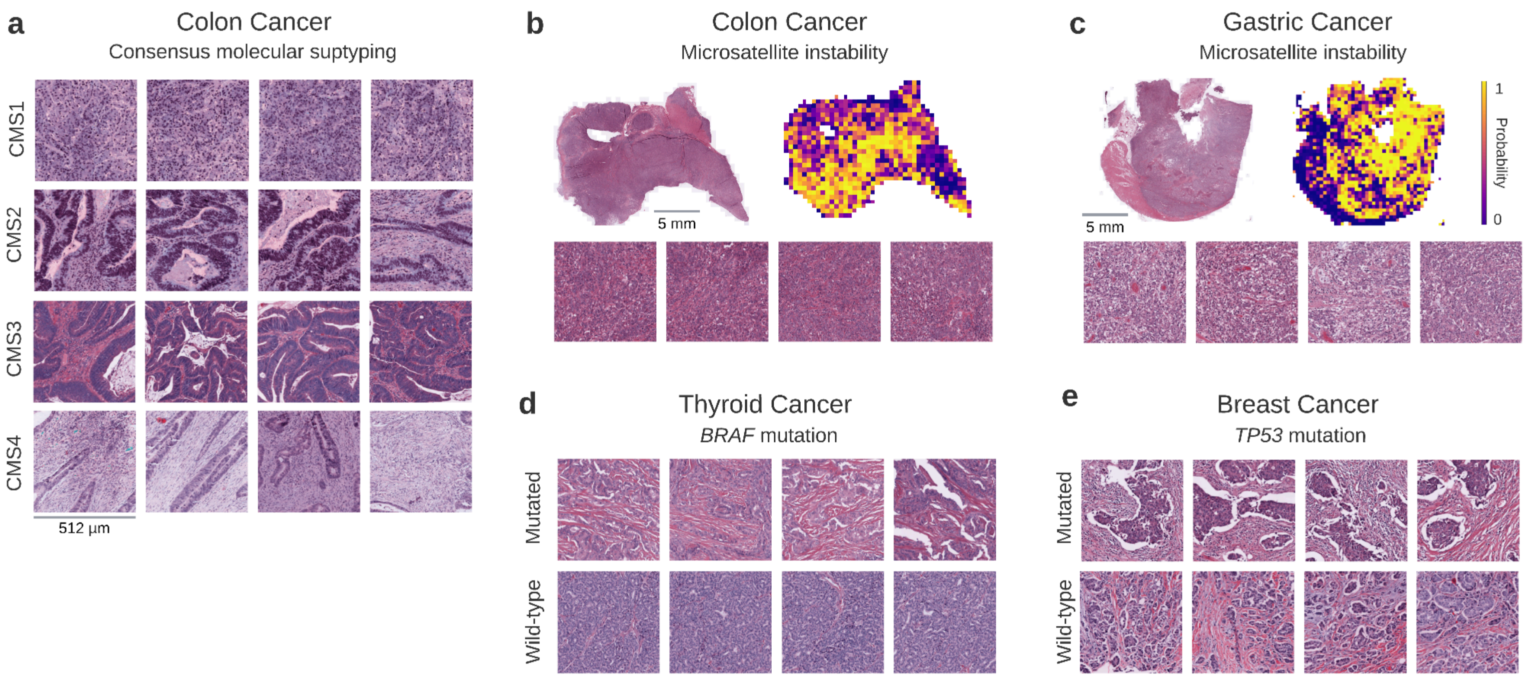

(a) The top-ranking tiles from the consensus molecular subtypes (CMS) of colon cancer (i.e. CMS1, CMS2, CMS3, and CMS4) show distinct morphological features. One can see lymphocytic infiltration patterns in CMS1, well-differentiated glandular structures for CMS2-3, and high stromal content in CMS4 tiles. **(b-c)** Highly predicted tiles from colon and gastric cancer patients showing morphological traits associated with microsatellite instability. **(d-e)**. The highest ranking tiles for the prediction of *BRAF* mutation in thyroid carcinoma **(d)** and *TP53* mutation in breast cancer **(e)** compared to their wild-type counterparts. Scale bar for slides: 5 mm. Scale bar for tiles: 512 µm.

Supplementary Fig. 9: Performance of individual biomarkers across selected cancer types

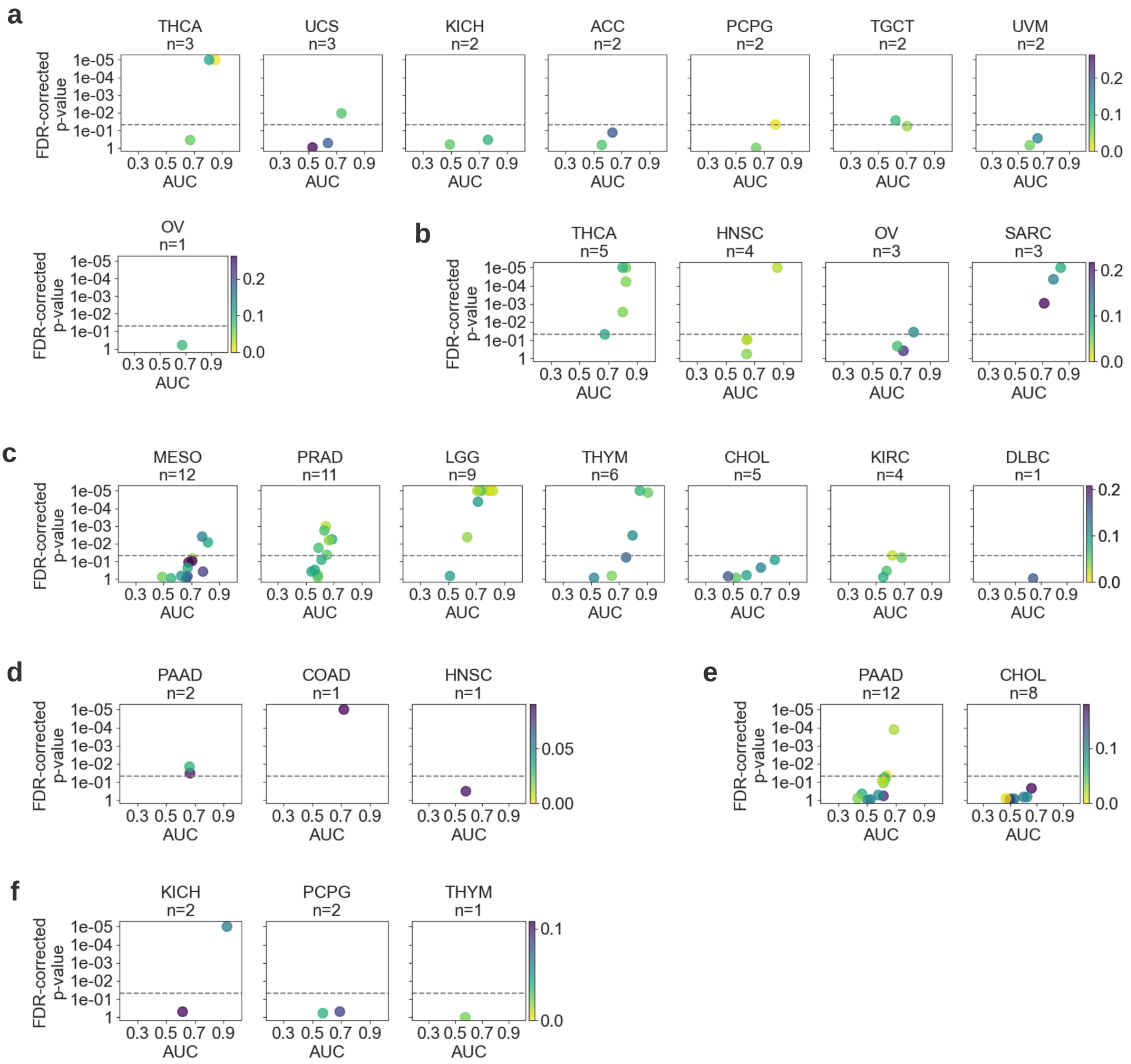

Scatter plots showing the performance of each model trained to predict biomarkers across different categories, namely, **(a)** the presence of single nucleotide variants in driver genes, **(b)** protein under-/over-expression status, **(c)** under-/over-expression of driver genes at the transcript level, **(d)** standard clinical biomarkers, **(e)** gene signatures, and subtypes, and finally, **(f)** clinical outcomes and treatment responses. Only the cancer types excluded from the main figures due to space limitations are shown here. Please refer to the caption of **Fig. 3** for a detailed explanation of the visualization. Cancer abbreviations are defined in Supplementary Table 1.

Supplementary Tables

Supplementary Table 1: Number of images and patients included in the study.

Abbr.	Cancer type	# images	# patients
LUSC	Lung squamous cell carcinoma	453	419
PRAD	Prostate adenocarcinoma	449	403
COAD	Colon adenocarcinoma	442	434
SKCM	Skin cutaneous melanoma	418	377
THCA	Thyroid carcinoma	504	492
OV	Ovarian serous cystadenocarcinoma	105	104
BRCA	Breast cancer	1061	992
STAD	Stomach adenocarcinoma	358	333
DLBC	Lymphoid neoplasm diffuse large B-cell lymphoma	38	38
BLCA	Bladder urothelial carcinoma	450	379
CHOL	Cholangiocarcinoma	38	38
PAAD	Pancreatic adenocarcinoma	204	180
UCEC	Uterine corpus endometrial carcinoma	509	448
KIRP	Kidney renal papillary cell carcinoma	269	245
LUAD	Lung adenocarcinoma	512	449
CESC	Cervical squamous cell carcinoma	265	255
KIRC	Clear cell renal cell carcinoma	499	493
THYM	Thymoma	179	120
LIHC	Liver hepatocellular carcinoma	369	361
SARC	Sarcoma	582	239
HNSC	Head and neck squamous cell carcinoma	434	414
READ	Rectum adenocarcinoma	158	157
ESCA	Esophageal carcinoma	157	155
LGG	Brain lower grade glioma	823	472
KICH	Kidney chromophobe	120	108
GBM	Glioblastoma multiforme	666	233
MESO	Mesothelioma	81	70
PCPG	Pheochromocytoma and paraganglioma	193	174
TGCT	Testicular germ cell tumors	253	148
UCS	Uterine carcinosarcoma	87	53
UVM	Uveal melanoma	53	53
ACC	Adrenocortical carcinoma	225	54
	Total:	10954	8890

Supplementary Table 2: Number of images and patients included in the CPTAC dataset.

Abbr.	TCGA-like abbr.	Cancer type	# images	# patients
UCEC	UCEC	Uterine corpus endometrial carcinoma	591	247
PDA	PAAD	Pancreatic ductal adenocarcinoma	382	168
LSCC	LUSC	Lung squamous cell carcinoma	689	211
LUAD	LUAD	Lung adenocarcinoma	669	224
HNSCC	HNSC	Head-and-neck cancer	268	112
GBM	GBM	Glioblastoma multiforme	510	189
COAD	COAD	Colon adenocarcinoma	372	178
		Total:	3481	1329

Only the cancers that have comparable biomarkers in the TCGA dataset were considered. The abbreviations used in the CPTAC and TCGA resources for the same cancer type are provided in the first two columns, respectively.

Supplementary Table 3: Details of the binarization rules used to define actionable survival outcomes.

Endpoint	Description	Binarization Rule
OS	Overall survival	Positive: Death from any cause. Negative: Alive.
DSS	Disease-specific survival	Positive: If vital_status is Dead and tumor_status is WITH TUMOR. If a patient died from the disease shown in the field of cause_of_death, the status of DSS would be Positive. Negative: If vital_status is Alive or vital_status is Dead and tumor_status is TUMOR FREE.
DFI	Disease-free interval	Positive: If a patient has a new tumor event whether it is a local recurrence, distant metastasis, new primary tumor of the cancer, including cases with a new tumor event whose type is N/A. Negative: First, treatment_outcome_first_course is "Complete Remission/Response"; if the tumor type doesn't have "treatment_outcome_first_course" then it is defined by the value "R0" in the field of "residual_tumor"; otherwise, it is defined by the value "negative" in the field of "margin_status".
PFI	Progression-free interval	Positive: If a patient has a new tumor event whether it is a progression of disease, local recurrence, distant metastasis, new primary tumors at all sites, or died with cancer without a new tumor event, including cases with a new tumor event whose type is N/A. Negative: Otherwise.

The information in this table is originally provided in the Integrated TCGA Pan-Cancer Clinical Data Resource[1] and is summarized here.

Supplementary Table 4: Average performance and standard deviation for all cancer types.

Abbr.	Cancer type	Mean AUC	Std. of AUCs
THCA	Thyroid carcinoma	0.768	0.091
TGCT	Testicular Germ Cell tumors	0.711	0.122
GBM	Glioblastoma multiforme	0.697	0.147
KIRP	Kidney renal papillary cell carcinoma	0.697	0.116
MESO	Mesothelioma	0.692	0.137
LGG	Brain Lower Grade Glioma	0.684	0.118
KIRC	Kidney renal clear cell carcinoma	0.679	0.111
ACC	Adrenocortical carcinoma	0.676	0.151
PCPG	Pheochromocytoma and Paraganglioma	0.670	0.086
STAD	Stomach adenocarcinoma	0.663	0.114
KICH	Kidney Chromophobe	0.661	0.143
BRCA	Breast invasive carcinoma	0.658	0.100
LIHC	Liver hepatocellular carcinoma	0.653	0.105
ESCA	Esophageal carcinoma	0.653	0.114
COAD	Colon adenocarcinoma	0.645	0.109
UCEC	Uterine Corpus Endometrial Carcinoma	0.645	0.101
PRAD	Prostate adenocarcinoma	0.644	0.092
SARC	Sarcoma	0.640	0.108
OV	Ovarian serous cystadenocarcinoma	0.636	0.137
HNSC	Head and Neck squamous cell carcinoma	0.634	0.104
SKCM	Skin Cutaneous Melanoma	0.632	0.115
BLCA	Bladder Urothelial Carcinoma	0.628	0.104
LUAD	Lung adenocarcinoma	0.627	0.111
PAAD	Pancreatic adenocarcinoma	0.626	0.118
CESC	Cervical squamous cell carcinoma	0.622	0.110
LUSC	Lung squamous cell carcinoma	0.613	0.103
CHOL	Cholangiocarcinoma	0.594	0.137
READ	Rectum adenocarcinoma	0.593	0.133
UCS	Uterine Carcinosarcoma	0.585	0.158

DLBC, UVM, and THYM were excluded from the table due to only constituting one to seven valid targets across all biomarker types. The abbreviations used for each cancer type are given in the first column.

Supplementary References

[1] Liu, J. et al. An Integrated TCGA Pan-Cancer Clinical Data Resource to Drive High-Quality Survival Outcome Analytics. Cell 173, 400-416.e11 (2018).
