# Supplementary material for: A systematic pan-cancer study on deep learning-based prediction of multi-omic biomarkers from routine pathology images
Source: Commun Med (Lond). 2024 Mar 15;4:48. doi: 10.1038/s43856-024-00471-5 (PMC10942985; doi:10.1038/s43856-024-00471-5)
Supplement: Supplementary file 2 — Description of Additional Supplementary Files [file 43856_2024_471_MOESM2_ESM.pdf]

# Description of Additional Supplementary Files

**File Name:** Supplementary Data 1
**Description:** AUC values, class prevalence, validation sample size, corrected p-values, and other relevant information for all models evaluated in the study.
