# Supplementary material for: A systematic pan-cancer study on deep learning-based prediction of multi-omic biomarkers from routine pathology images
Source: Commun Med (Lond). 2024 Mar 15;4:48. doi: 10.1038/s43856-024-00471-5 (PMC10942985; doi:10.1038/s43856-024-00471-5)
Supplement: Supplementary file 4 — Reporting Summary [file 43856_2024_471_MOESM4_ESM.pdf]

nature portfolio

Corresponding author(s): Salim Arslan

Last updated by author(s): Feb 29, 2024

Reporting Summary

Nature Portfolio wishes to improve the reproducibility of the work that we publish. This form provides structure for consistency and transparency in reporting. For further information on Nature Portfolio policies, see our Editorial Policies and the Editorial Policy Checklist.

Statistics

For all statistical analyses, confirm that the following items are present in the figure legend, table legend, main text, or Methods section.

n/a	Confirmed	
☐	☒	The exact sample size (*n*) for each experimental group/condition, given as a discrete number and unit of measurement
☒	☐	A statement on whether measurements were taken from distinct samples or whether the same sample was measured repeatedly
☐	☒	The statistical test(s) used AND whether they are one- or two-sided<br>*Only common tests should be described solely by name; describe more complex techniques in the Methods section.*
☒	☐	A description of all covariates tested
☐	☒	A description of any assumptions or corrections, such as tests of normality and adjustment for multiple comparisons
☐	☒	A full description of the statistical parameters including central tendency (e.g. means) or other basic estimates (e.g. regression coefficient) AND variation (e.g. standard deviation) or associated estimates of uncertainty (e.g. confidence intervals)
☐	☒	For null hypothesis testing, the test statistic (e.g. *F*, *t*, *r*) with confidence intervals, effect sizes, degrees of freedom and *P* value noted<br>*Give P values as exact values whenever suitable.*
☒	☐	For Bayesian analysis, information on the choice of priors and Markov chain Monte Carlo settings
☒	☐	For hierarchical and complex designs, identification of the appropriate level for tests and full reporting of outcomes
☐	☒	Estimates of effect sizes (e.g. Cohen's *d*, Pearson's *r*), indicating how they were calculated

Our web collection on statistics for biologists contains articles on many of the points above.

Software and code

Policy information about availability of computer code

Data collection	Data in this study was provided by TCGA and CPTAC. OpenSlide python library (openslide-python) version 1.1.1 was used to read whole slide images.
Data analysis	The experiments and analyses in this study were carried out with python 3.6 and the following python libraries: torch (1.9.0), torchvision (0.10.0), google-cloud-storage (1.32.0), openslide-python (1.1.1), pillow (6.0.0), opencv (3.4.2), tensorboard (1.15.0), numpy (1.18.1), pandas (1.3.5), seaborn (0.11.0), scipy (1.4.1), scikit-learn (0.22.1), statsmodels (0.11.0), and matplotlib (3.5.1)

For manuscripts utilizing custom algorithms or software that are central to the research but not yet described in published literature, software must be made available to editors and reviewers. We strongly encourage code deposition in a community repository (e.g. GitHub). See the Nature Portfolio guidelines for submitting code & software for further information.

Data

Policy information about availability of data

All manuscripts must include a data availability statement. This statement should provide the following information, where applicable:

- Accession codes, unique identifiers, or web links for publicly available datasets
- A description of any restrictions on data availability
- For clinical datasets or third party data, please ensure that the statement adheres to our policy

TCGA whole slide images are available at https://portal.gdc.cancer.gov/. Genetic, transcriptomic, proteomic, and clinical data used to generate biomarker profiles

for cases in the TCGA cohorts are available at https://portal.gdc.cancer.gov/ and https://cbioportal.org/. Clinically relevant driver genes are available at https://cancervariants.org/. CPTAC whole slide images are available at https://wiki.cancerimagingarchive.net/display/Public/CPTAC+Imaging+Proteomics/. Genetic data used to generate biomarker profiles for cases in the CPTAC cohorts are available at https://portal.gdc.cancer.gov/. The source data used to generate the figures, including AUC values, class prevalence, validation sample size, corrected p-values, and the other relevant information for the models evaluated in the study, are given in Supplementary Data 1.

Human research participants

Policy information about studies involving human research participants and Sex and Gender in Research.

Reporting on sex and gender	N/A
Population characteristics	TCGA and CPTAC cohorts include data from a diverse population collected across multiple sites. All information about the participants are available at https://portal.gdc.cancer.gov/.
Recruitment	The authors had no role in the recruitment of participants.
Ethics oversight	Only retrospective data was used in this study, without any active involvement of patients. Ethics oversight of the TCGA study is described at https://www.cancer.gov/about-nci/organization/ccg/research/structural-genomics/tcga/history/policies

Note that full information on the approval of the study protocol must also be provided in the manuscript.

Field-specific reporting

Please select the one below that is the best fit for your research. If you are not sure, read the appropriate sections before making your selection.

☒ Life sciences ☐ Behavioural & social sciences ☐ Ecological, evolutionary & environmental sciences

For a reference copy of the document with all sections, see nature.com/documents/nr-reporting-summary-flat.pdf

Life sciences study design

All studies must disclose on these points even when the disclosure is negative.

Sample size	No sample size calculations were performed. We used the publicly-available TCGA and CPTAC datasets. The TCGA dataset consisted of 10,954 diagnostic slides of 8,890 patients. Only images scanned at 0.5 microns per pixel (MPP) were kept and images with no MPP information were automatically discarded. The number of the images and patients included in the TCGA cohort are provided in Supplementary Table 1 and further details are given in Methods: Dataset. The CPTAC dataset consisted of 3,481 images corresponding to 1,329 patients. Only images scanned at 0.5 MPP were used. The details of the final images and patients included in the CPTAC cohort are provided in Supplementary Table 2 and Methods: External dataset.
Data exclusions	Data were excluded based on the following pre-established exclusion criteria:<br>- TCGA: Slides that were not scanned at 0.5 microns per pixel (MPP) were discarded as indicated in Methods: Dataset.<br>- CPTAC: Slides with a resolution different than 0.25 MPP in COAD and 0.5 MPP in other datasets were discarded as indicated in Methods: External dataset.<br>- Slides with missing biomarker profiles were discarded as indicated in Methods: Biomarker acquisition.
Replication	Attempts to reproduce the performance metrics from the saved model weights were successful. Raw performance metrics used to derive the results, visualizations, and plots are given in Supplementary Data 1.
Randomization	Experiments were done in 3-fold cross-validation setting, where the cases with a valid biomarker status in each dataset were split into three random partitions (folds), each having approximately the same proportion of positive samples. The images were partitioned at the patient level so that no patient could appear in multiple folds.
Blinding	Blinding was not relevant because our study is based on retrospective analysis of publicly-available histology images.

Reporting for specific materials, systems and methods

We require information from authors about some types of materials, experimental systems and methods used in many studies. Here, indicate whether each material, system or method listed is relevant to your study. If you are not sure if a list item applies to your research, read the appropriate section before selecting a response.

Materials & experimental systems

n/a	Involved in the study
☒	☐ Antibodies
☒	☐ Eukaryotic cell lines
☒	☐ Palaeontology and archaeology
☒	☐ Animals and other organisms
☒	☐ Clinical data
☒	☐ Dual use research of concern

Methods

n/a	Involved in the study
☒	☐ ChIP-seq
☒	☐ Flow cytometry
☒	☐ MRI-based neuroimaging
